# Adipocyte heparan sulfate determines type 2 diabetes susceptibility in mice via FGF1-Mediated glucose regulation

**DOI:** 10.1016/j.molmet.2025.102267

**Published:** 2025-10-08

**Authors:** Chung-Jui Yu, Ariane R. Pessentheiner, Sihao Liu, Sarah Wax, Marissa L. Maciej-Hulme, Chelsea D. Painter, Bastian Ramms, Daniel R. Sandoval, Anthony Quach, Natalie DeForest, G. Michelle Ducasa, Chiara Tognaccini, Caroline Labib, Norah Al-Azzam, Friederike Haumann, Greg Trieger, Patrick Secrest, Amit Majithia, Aaron C. Petrey, Kamil Godula, Annette R. Atkins, Michael Downes, Ronald M. Evans, Philip L.S.M. Gordts

**Affiliations:** 1 Department of Medicine, Division of Endocrinology and Metabolism, University of California, San Diego, CA, USA; 2 Department of Pathology, Division of Microbiology & Immunology, University of Utah, School of Medicine, Salt Lake City, UT, USA; 3 University of Utah Molecular Medicine Program, University of Utah, School of Medicine, Salt Lake City, UT, USA; 4 Institute of Molecular Biosciences, Division of Biophysics, University of Graz, Austria; 5 Gene Expression Laboratory, Salk Institute for Biological Studies, La Jolla, CA, USA; 6 Department of Chemistry and Biochemistry, University of California, San Diego, CA, USA; 7 Department of Biochemistry, University Medical Center Hamburg-Eppendorf, Hamburg, Germany; 8 Glycobiology Research and Training Center, University of California, San Diego, CA 92093, USA

**Keywords:** Type 2 diabetes, FGF1, Heparan Sulfate, Adipocyte, Extracellular Matrix, Glycocalyx

## Abstract

Obesity is the principal driver of insulin resistance, and lipodystrophy is also linked with insulin resistance, emphasizing the vital role of adipose tissue in glucose homeostasis. The quality of adipose tissue expansion is a critical determinant of insulin resistance predisposition, with individuals suffering from metabolic unhealthy adipose expansion exhibiting greater risk. Adipocytes are pivotal in orchestrating metabolic adjustments in response to nutrient intake and cell intrinsic factors that positively regulate these adjustments are key to prevent Type-2 diabetes. Employing unique genetic mouse models, we established the critical involvement of heparan sulfate (HS), a fundamental element of the adipocyte glycocalyx, in upholding glucose homeostasis during dietary stress. Genetic models that compromise adipocyte HS accelerate the development of high-fat diet-induced hyperglycemia and insulin resistance, independent of weight gain. Mechanistically, we show that perturbations in adipocyte HS disrupts endogenous FGF1 signaling, a key nutrient-sensitive effector. Furthermore, compromising adipocyte HS composition detrimentally impacts FGF1-FGFR1-mediated endocrinization, with no significant improvement observed in glucose homeostasis. Our data establish adipocyte HS composition as a determinant of Type 2 diabetes susceptibility and the critical dependency of the endogenous adipocyte FGF1 metabolic pathway on HS.

## HIGHLIGHTS

• Adipocyte heparan sulfate does not impact diet-induced weight gain.
• Adipocyte heparan sulfate sulfation compromises glucose regulation and insulin sensitivity under nutrient stress.
• Mice with reduced HS sulfation show increased insulin resistance and fatty liver disease in a diet-induced obesity model.
• HS sulfation is essential for FGF1 glucose-lowering, a critical paracrine insulin sensitizer in adipose tissue.

## Graphical abstract

**Figure:**
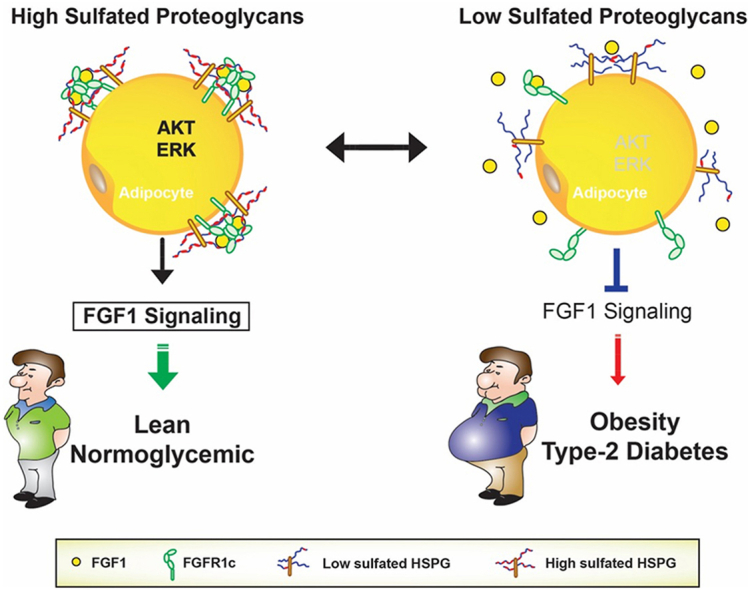

## 1 Introduction

Type-2 diabetes (T2D) is a metabolic disorder of global magnitude, and originates from multi-organ insulin resistance, which amplifies the demand for insulin to eliminate glucose from the circulation [1]. The compensatory hyperinsulinemia progressively diminishes due to β-cell failure necessitating therapeutic insulin administration. Obesity is the leading cause of insulin resistance, and conversely lipodystrophy is also associated with insulin resistance, supporting a central role for adipose tissue (AT) in glucose homeostasis [[2], [3], [4], [5], [6], [7]]. The risk to develop T2D increases linearly with an increase in body mass index [2]. However, the functionality of AT expansion, rather than its quantity, is a key factor in predisposition to insulin resistance [8]. Patients with metabolic unhealthy obesity possess up to 20-fold greater risk of developing T2D compared to metabolic healthy obesity [9]. Such observation implies that natural variation in the quality of the adipocyte's intrinsic metabolic adaptive responses contribute to variations in T2D onset and health outcomes in obese individuals [4,8,10].

Adipocytes adapt to nutritional excess by increasing the adipocyte size and cell number. To allow those changes, the glycocalyx and extracellular matrix (ECM) surrounding adipocytes undergoes constant remodeling [2]. The ECM components are critical regulators of the adaptive plasticity and metabolic response of adipocytes. Heparan sulfate (HS) proteoglycans (HSPG) are a group of key molecules found at the cell surface and in the glycocalyx and ECM of all cells, including adipocytes [11]. HSPGs consist of one or more covalently linked HS chains attached to a small group of 17 core proteins [11]. The HS chain is a disaccharide repeat unit of uronic acid and *N*-acetyl glucosamine, a process catalyzed by a heterodimeric complex of exostosin (EXT) 1 and EXT2. In the Golgi the HS chain undergoes extensive modifications with sequential *N*-deacetylation and *N*-sulfation catalyzed by the *N*-deacetylase/*N*-sulfotransferase (−4) enzymes. The initial sulfation of the amine group allows additional sulfation modifications and epimerization by 2-*O*, 3-*O*, and 6-*O*-sulfotransferases and C5-epimerase. This process increase the structural diversity of the HS chain [12], encoding regions of negatively charged patterns that are key elements in binding various positively charged ligands [12].

Pre-adipocyte HS is critical for their differentiation into mature adipocytes [11,13,14]. However, it is unclear how mature adipocyte-derived HS modulates endocrine responses during nutrient challenges [11]. To evaluate how compositional variation in adipocyte HS composition influence glucose metabolism we used mice lacking HS biosynthetic enzymes in AT specifically. Our results show that reduced HS sulfation and impaired HS production via *Ndst1* and *Ext1* inactivation in AT, respectively, promote glucose intolerance and insulin resistance by attenuating the endogenous FGF1-metabolic activity.

## 2 Materials and methods

### 2.1 Mice

*Ext1*^f/f^ Adiponectin-Cre ^*+*^ mice, *Fgfr1*^*f/f*^ Adiponectin-Cre ^*+*^ mice, and *Ndst1*^f/f^ Adiponectin-Cre ^*+*^ mice were generated by crossing the *Ext1*^f/f^ (gifted by Dr. Yu Yamaguchi), Fgfr1c (The Jackson Laboratory), and *Ndst1*^f/f^ (gifted by Dr. Jeffrey Esko) mice to AdipoQ-Cre transgenic mice (The Jackson laboratory) [15,16]. *Ndst1*^f/f^ and *Ndst1*^f/f^ Alb-Cre ^*+*^ mice were generated as described before [15]. Mice were kept on a 12-hour light cycle and were fed *ad libitum* with water and standard rodent chow (PicoLab® Rodent Diet 20 5053) or a high-fat diet (60% fat in calories, Research Diets D12492). Solutions of hFGF1 (0.1 mg/ml in PBS; Prospec) were injected as described. All animals were housed and bred in vivaria approved by the Association for Assessment and Accreditation of Laboratory Animal Care located in the School of Medicine, UCSD, following standards and procedures approved by the UCSD Institutional Animal Care and Use Committee.

### 2.2 Blood parameters

Whole blood was taken from facial vein and blood glucose was measured with a glucose meter (Accu-Chek Performa, Roche Diabetes Care ME) from the tail vein. Plasma was collected after centrifugation at 5000 rpm. Commercially available kits were used to determine plasma triacylglycerol (TG), cholesterol (Chol) (Sekisui Diagnostics) and free fatty acids (FFAs) (NEFA kit, WAKO) levels. Plasma insulin, leptin, and adiponectin levels were measured with Mouse Ultrasensitive Insulin ELISA and Mouse Adiponectin or Leptin ELISA Kits (Crystal Chem), respectively. Lipoprotein distribution was obtained by size exclusion via fast protein liquid chromatography (FPLC).

### 2.3 Body composition and indirect calorimetric measurements

Mice were placed into Comprehensive Lab Animal Monitoring System (CLAMS; Columbus Instruments). Rates of O2 consumption (VO2; ml/kg/h) and CO2 production (VCO2) were measured for each chamber every 17 min throughout the study.

### 2.4 Insulin (ITT), glucose (GTT) and pyruvate tolerance test (PTT)

Mice were fasted prior to ITT, GTT, and PTT for 5–6 h. 0.5 U/kg human insulin (Sigma) in 0.9% NaCl/0.8% BSA in HFD-fed mice or 0.35 U/kg insulin in chow-fed mice, and 2 g/kg sodium pyruvate in water were i.p. injected and blood glucose levels from tail vein were monitored after 15, 30, 60, 90, and 120 min. 2 g/kg d-glucose in water was gavaged and blood glucose levels were monitored 20, 40, 60, and 90 min. Blood for insulin measurements were taken prior to and 20 min after glucose gavage via facial vein bleeding.

### 2.5 *In vivo* mFGF1^WT^ metabolic studies

Recombinant murine wildtype mFGF1^WT^ was either obtained from Prospec or was a generous gift from Ronald Evans (obtained from ExonBio). mFGF1^WT^ was reconstituted in PBS and administered with the concentrations stated in figure legends and text. Generally, 0.5 mg/kg rFGF1 was administered to mice via intraperitoneal injection.

### 2.6 *In vivo* mFGF1^WT^ Pharmacological studies

Recombinant mFGF1^WT^ was diluted to 0.1 mg/mL in sterile-filtered PBS and delivered through intraperitoneal injection to mice fasted for 16 h at a 0.1 mg mFGF1^WT^ kg^−1^ fasted body weight dose. 20 min after injection, mice were sacrificed and gonadal white adipose tissue (gWAT) fat pads were dissected, flash frozen in liquid N_2_, and stored at −80 °C for future analysis.

### 2.7 Heparan sulfate purification and disaccharide analysis

AF and SVF or homogenates of total tissue were used for heparan sulfate isolation. Heparan sulfate purification and disaccharide mass spectrometry analysis were quantified as described as previously reported with modification described below [17]. Around 50–100 mg of adipose tissue was suspended in 1 mL tissue digestion buffer (1 mg/mL Pronase, 0.1% Triton X-100, 10 mM CaCl_2_, 50 mM Tris pH 7.6) and incubated at 60 °C for two days. Tissue digest was then diluted to 10 mL with 0.2 M NaCl, 20 mM sodium acetate pH 6 buffer. One mL DEAE resin (Sepheracel, GE Healthcare) was equilibrated in 10 mL of 0.2 M NaCl, 0.1% Triton X-100, 50 mM sodium acetate pH 6 buffer. Tissue digest was loaded onto the column, washed with 20 mL of 0.2 M NaCl, 20 mM sodium acetate pH 6 buffer, and eluted with 2.5 mL of 2 M NaCl, 20 mM sodium acetate pH 6 buffer. Elutions were desalted by gel filtration (PD-10, Cytiva) and lyophilized. DNase treatment was performed by resuspending lyophilized samples in 500 μL of 20 kU/mL DNase I, 0.5 mM CaCl_2_, 5 mM MgCl_2_, 50 mM NaCl, 50 mM Tris pH 8 buffer and incubated at 37 °C for 2 h. To release heparan sulfate chains from their protein cores by β-elimination, solutions after DNase digestion were then brought to 0.4 M NaOH and incubated overnight at 4 °C. Sample solutions were subsequently neutralized with 10 M acetic acid, then diluted by 20-fold in 0.2 M NaCl, 20 mM sodium acetate pH 6 buffer. Heparan sulfate glycosaminoglycans (HS-GAG) were re-purified over DEAE anion-exchange chromatography, desalted by gel filtration, and lyophilized as described.

Lyophilized samples were resuspended in heparinase buffer consisting of 2 mU heparinase I, II, and III (IBEX Pharmaceuticals), 40 mM ammonium acetate, 3.3 mM calcium acetate pH 7. Samples were incubated at 37 °C for 3 h, then dried overnight in a vacuum concentrator. Dried samples were resuspended in 17 uL aniline, followed by addition of 17 uL of 1.2 M sodium cyanoborohydride, 30% glacial acetic acid in DMSO reductant solution. Samples were briefly vortexed, then incubated at 37 °C for 16 h. Samples were dried overnight in a vacuum concentrator. Aniline-tagged samples were dissolved in 20 μL LC/MS-grade water (Fisher Scientific). 5 μL of the sample was mixed with 2 μL 10X LC-MS buffer A (50 mM dibutylamine, 80 mM acetic acid), 2 μL LC/MS-grade water, and 1 μL ^13^C_6_-aniline tagged HS disaccharide standards. LC-MS analysis was conducted on a Thermo Fisher LTQ-Orbitrap in negative mode with TARGA C18 reverse phase column (Higgins Analytical TS-1501-C185). Isocratic steps during the separation are as follows: 100% LC-MS buffer A (5 mM dibutylamine, 8 mM acetic acid) for 10 min, 17% LC-MS buffer B (5 mM dibutylamine, 8 mM acetic acid in 70% methanol) for 10 min, 32% LC-MS buffer B for 15 min, 40% LC-MS buffer B for 15 min, 50% LC-MS buffer B for 15 min, 60% LC-MS buffer B for 15 min, 100% LC-MS buffer B for 10 min, and 100% LC-MS buffer A for 10 min. Data analysis was conducted in Thermo Fisher Xcalibur software. Total ion chromatograms for each aniline-tagged HS disaccharide and its corresponding ^13^C_6_-aniline tagged analogue was integrated from full width at half maximum.

### 2.8 Recombinant murine FGF1 cloning and production

Vector pMCSG53-DsbC was a gift from Alexei Savchenko (Addgene plasmid # 186623; http://n2t.net/addgene:186623; RRID:Addgene_186623). Insert gene fragment encoding murine FGF1 (mFGF1^WT^, UniProt P61148, residues 16–155) with N-terminal polyhistidine (6xHis) tag and Tobacco Etch Virus (TEV) cleavage site (ENLYFQS) was codon optimized and synthesized from Integrated DNA Technologies. Insert gene was cloned into vector pMCSG53-DsbC with NEB Gibson Assembly Master Mix. Non–HS–binding mFGF1 (mFGF1^ΔHBS^) was generated from mFGF1 vector assembly with site-directed mutagenesis at the following positions: K127D, K128Q, and K133V. All assemblies were verified with Sanger DNA sequencing at Genewiz from Azenta Life Sciences. Vector expression was performed in NEB® 5-alpha F’*I*^*q*^ competent *E. coli* cells and purified with Promega Wizard® *Plus* SV Minipreps kit. Protein expression was conducted as described with modifications [18]. Plasmids encoding mFGF1^WT^ or mFGF1^ΔHBS^ were transformed in ClearColi® BL21(DE3) electrocompetent cells according to manufacturer protocols, then plated on Luria Bertani (LB) agar plates supplemented with 100 μg/mL carbenicillin and incubated overnight at 37 °C. 50 mL of autoclaved LB broth supplemented with 100 μg/mL carbenicillin in a shaker flask was inoculated with a single colony from the agar plate and grown at 37 °C with 200 rpm shaking overnight. This overnight culture was diluted to 1 L of LB broth with 100 μg/mL carbenicillin and grown at 37 °C with 200 rpm shaking. Expression was induced with 0.6 mM IPTG when optical density (OD600) reaches 0.6–0.8 AU, then allowed to incubate at 17 °C overnight. These bacterial cultures were collected by centrifugation at 4500×*g* and stored at −80 °C until purification. All purification steps were conducted at 4 °C within 2 days of cell pellet lysis. Cell pellets were resuspended in 25 mL binding buffer (500 mM NaCl, 5% v/v glycerol, 100 mM HEPES pH 7.5) with 1 mg/mL lysozyme (Sigma–Aldrich L6876) and incubated at room temperature with stirring for 30 min. Cell suspensions were lysed by sonication in an ice bath (15 s on, 45 s off, 15 min total sonication time). The lysates were centrifuged at 4500×*g* for 15 min, then filtered through a 0.22 μm filter to remove cell debris. The resulting lysates were purified on a 1 mL HisTrap HP Ni-NTA column (Cytiva 17524801) attached to a peristaltic pump as follows: load lysates onto Ni-NTA column, washed with 12 mL of binding buffer, 2 × 12 mL of wash buffer (30 mM imidazole, 500 mM NaCl, 5% v/v glycerol, 100 mM HEPES pH 7.5), and eluted with 6 mL of elution buffer (250 mM imidazole, 500 mM NaCl, 5% v/v glycerol, 100 mM HEPES pH 7.5). The elution fraction was concentrated to 1 mL with Pierce™ 10 kDa MWCO Protein Concentrator, then purified through size-exclusion chromatography (Superdex 200) on ÄKTA pure™ FPLC system with phosphate-buffered saline (PBS). Purity of all recombinant proteins were evaluated with SDS-polyacrylamide gel electrophoresis. Protein concentrations were determined with a Thermo Scientific™ NanoDrop™ 2000c Spectrophotometer. Purified proteins were stored in 15 μM aliquots, flash frozen in liquid N_2_, and stored at −80 °C.

### 2.9 Cell-surface FGF1 binding analysis by flow cytometry

To assess FGF1 binding to WT or *Ndst1*^*−/−*^ mouse embryonic fibroblasts (MEFs), cells were lifted with 10 mM EDTA in DPBS and treated with 4 mU/mL heparan sulfate lyase I, II, III (HepLyase) in serum-free media for 30 min at 37 °C. 150,000 cells were resuspended in 1% BSA in DPBS and incubated with 10 μg/mL mFGF1^WT^ for 60 min at 4 °C. After incubation, the cells were washed three times with cold DPBS and centrifugation for 2 min at 400×*g*. The cells were then incubated with THE™ His Tag Antibody [iFluor 488], mAb for 60 min at 4 °C and washed three times as described previously before analysis on a FACSCalibur (BD Biosciences). Mean fluorescence intensity was assessed after adjusting forward and side scatter and gating for live cells. Analysis was done with FlowJo Analytical Software (Tree Star Inc.). The extent of protein binding was quantified by the geometric mean of the fluorescence intensity.

### 2.10 Adipocytes and stromal vascular cell isolation

Around 1g subcutaneous WAT from 8 to 14-weeks old male or female mice was dissected, washed, minced, and digested in 1 mL DMEM containing 0.25 U/mL collagenase D (Sigma) at 37 °C with constant agitation at 200 rpm for 25–35 min. To stop the digestion, complete DMEM including 10% FBS and 1% P/S was added to the digestion mixture. Then the cells were filtered through a 100 μm cell strainer. After centrifugation at 200×*g* for 10 min, the floating adipocyte fraction (AF) and the pellet containing stromal vascular fraction (SVF) were collected for HS, protein and mRNA isolation.

### 2.11 Cell culture and adipogenic differentiation

All cell culture flasks and plates were incubated at 37 °C in a humidified atmosphere with 5% CO_2_. 3T3-L1 murine adipocytes were purchased from Zen-Bio (SP-L1-F). Wild-type (WT) mouse embryonic fibroblasts (MEFs) and *Ndst1*^−/−^ MEFs [14]. Iron-fortified bovine calf serum (BCS) was purchased from ATCC (30–2030). Fetal bovine serum (FBS) was purchased from Omega Scientfic, Inc. (FB-01). Gibco™ Dulbecco's modified Eagle's medium, high glucose (DMEM, 11965092), Penicillin-Streptomycin 10,000 U/mL (Pen-Strep, 15140122), 0.25% Trypsin-EDTA (25200056), and Dulbecco's PBS (14190144) were purchased from ThermoFisher Scientific. Corning™ Costar™ flat bottom cell culture plates were purchased from Fisher Scientific. T225 TC treated cell culture flasks were purchased from ThermoFisher Scientific (159934). Dexamethasone (D4902), 3-isobutyl-1-methylxanthine (IBMX, I5879), rosiglitazone (R2408), and human insulin solution (I9278) were purchased from Sigma–Aldrich. MEFs [14] and SVF cells were seeded on a 24-well cell culture plate at a density of 30,000 cells/cm^2^. Cells were allowed to grow to confluence for 48 h in DMEM supplemented with 10% FBS containing FFA and 1% Pen-Strep. At this point (Day 0), the media were removed, cells were washed with PBS, and differentiation media with or without heparin (100 μg/ml) were added. Differentiation media consisted of 0.1 μM dexamethasone, 450 μM 3-isobutyl-1-methylxanthine, 2 μM insulin, and 1 μM rosiglitazone in DMEM supplemented with 10% FBS containing FFA and 1 × Pen-Strep (Sigma). On day 3 of differentiation, cells were washed with PBS and treated with insulin media, which consisted of 2 μM insulin, and 1 μM rosiglitazone in DMEM with 10% FBS containing FFA and 1 × Pen-Strep. 3T3-L1 preadipocyte cells were cultured in T225 culture flasks seeded at a density of 2,000 cells/cm^2^ in Culture Media (CM): DMEM supplemented with 10% BCS and 1% Pen-Strep. Cells were passaged by incubation in 5 mL of 0.25% Trypsin-EDTA at 37 °C for 2 min once the cells reached 80–90% confluency. Subculture cell samples were frozen in a media mixture of 90% BCS DMEM media and 10% DMSO. Cell assay plates were seeded at a density of 60,000 cells/cm^2^ in CM media. Cells were allowed to grow to confluence for 24 h, then incubated for an additional 48 h to fully arrest growth. On day 3 after seeding, wells were washed twice with DMEM supplemented with 10% FBS and 1% Pen-Strep, then differentiation media 1 was added to each well. Differentiation media 1 consisted of 1 μM dexamethasone, 500 μM IBMX, 2 μM rosiglitazone, and 0.96 μM human insulin in 10% FBS DMEM media. On day 5, the wells were washed twice with 10% FBS DMEM media, then differentiation media 2 was added to each well. Differentiation media 2 consists of 0.96 μM human insulin in 10% FBS DMEM media. On day 7, wells were washed twice with 10% FBS DMEM media and maintenance media was added to each well. Maintenance media consists of 10% FBS DMEM media. Media in each well was changed every 2–3 days until the cells reached day 9–12 (day 12–15 after seeding). All experiments with cell assay plates were conducted within this time frame.

### 2.12 Analytical heparin-sepharose chromatography

FGF1, FGF19, or FGF21 was applied to a 1 ml HiTrap Heparin Sepharose column (GE Healthcare) in PBS. Protein was eluted with a gradient of NaCl from 150 mM to 2 M.

### 2.13 Gene expression analysis

Total RNA from homogenized tissue and cells was isolated and purified using E.Z.N.A. HP Total RNA (Omega) or RNeasy mini (Qiagen) kits according to the manufacturer's instructions. On-column DNA removal was performed with RNase-free DNase set (Qiagen). The quality and quantity of the total RNA was monitored and measured with NanoDrop (NanoDrop Technologies, Inc. Wilmington, DE). cDNA was synthesized from the RNA samples using SuperScript™ III First-Strand Synthesis System (Invitrogen™) according to manufacturer's protocols. 5–10 ng of cDNA was used for quantitative real-time PCR with gene-specific primers (**Data** Table S1) and TBP as a house keeping gene on a BioRad CFX96 Real-time PCR system (Bio Rad).

### 2.14 Western Blot protein expression analysis

Cells or tissue were homogenized with RIPA buffer supplemented with EDTA-free proteinase inhibitor cocktail (PIC, Sigma) and PhosSTOP (Roche). In the event DNA contamination is significant, tissues were homogenized in sucrose lysis buffer (250 mM sucrose, 1 mM EGTA, 5 mM Tris pH 7.4) with proteinase and phosphatase inhibitor cocktails. Cell or tissue lysates were incubated on ice for 30 min, then centrifuged at 16,000 g, 4 °C for 15 min and the aqueous middle layer was collected and frozen at −20 °C until usage. Protein concentrations were determined with the BCA protein assay kit (Pierce). Protein samples were diluted in sodium dodecyl sulfate (SDS) and dithiothreitol (DTT) loading buffer and boiled for 5 min at 95 °C. Proteins were resolved by SDS-polyacrylamide gel electrophoresis and transferred to PVDF membranes. The membranes were blocked 1 h at room temperature with Odyssey PBS Blocking Buffer (Li-Cor, 927–40000) or fish serum blocking buffer (Thermo Fisher, 37527). Individual proteins were detected with the specific antibodies against p-ERK (Cell Signaling Technology 9101, 1:1000), ERK (Cell Signaling Technology 4696, 1:2000), p-AKT (Cell Signaling Technology 4606, 1:2000) and AKT (Cell Signaling Technology 2920, 1:2000) in 5% w/v nonfat dry milk in TBST. The immunoreactive bands were blotted with IRDye® 800CW or 680RD secondary antibodies (LI-COR Biotech). Blots were imaged using an Odyssey Infrared Imaging System (LI-COR Biotech) and quantified using the ImageJ software.

### 2.15 Phospho-ERK1/2 homogenous time resolved fluorescence

HTRF Phospho-ERK1/2 (Thr202/Tyr204) detection kits were purchased from Revvity. All HTRF samples were prepared in HTRF 96-well low volume white plates (Revvity 66PL96005). Fluorescence measurements were conducted on Tecan Spark® and Revvity VICTOR Nivo multimodal plate reader. *In Vitro Adipocyte Cell Assays*: Day 9–12 differentiated 3T3-L1 adipocytes in 96 well cell culture plates were washed twice with 200 μL of serum-free DMEM media, then serum starved for 3 h at 37 °C. If applicable, removal of cell surface HS was performed by treatment of cells with 1 mU heparinase I, II, and III 30 min prior to the end of the serum starve. At the end of the serum starve, media in each well was replaced with new serum-free media with 1 mU heparinase I, II, and III (if applicable) and recombinant mFGF1^WT^ or mFGF1^ΔHBS^ produced from ClearColi, followed by incubation at 37 °C for 5 min. At the end of stimulation, the media in each well was rapidly aspirated. The cells were lysed and their phospho-ERK1/2 signals were measured according to manufacturer protocols. All signals were normalized to lysate total protein content determined by BCA assay. *Murine gWAT Tissue Assays*: 50–100 mg of dissected gWAT tissue were lysed with 250 μL of cold lysis buffer (150 mM NaCl, 10% glycerol, 1% Triton X-100, 1% sodium deoxycholate, 50 mM HEPES pH 7.5) by homogenization by bead-beater for 1 min. Samples were incubated on a rocker at 4 °C for 30 min, then centrifuged at 18,000×*g* for 30 min at 4 °C. The clear aqueous fraction below the fat cake was separated into new tubes. Phospho-ERK1/2 signals were measured with the lysates according to the manufacturer protocols. All signals were normalized to lysate total protein content determined by BCA assay.

### 2.16 Histology

Tissue samples were fixed in 10% buffered formalin and embedded in paraffin. Sections were stained with haematoxylin and eosin (H&E) according to standard protocols. Adipocyte size was assessed with ImageJ software. At least 3 different areas per individual section per mouse fat pad were analyzed at 200x magnification.

### 2.17 Statistical analysis

If not otherwise stated results are mean values ± SEM of at least three independent experiments or mice or results show one representative experiment out of three. Statistical analysis was done on all available data. Statistical significance was determined using the 2-tailed student's t-test, one-way ANOVA followed by a Bonferroni post hoc test or two-way ANOVA to compare time courses. For statistical analysis GraphPad prism 7 software was used. ∗p < 0.05, ∗∗p < 0.01, ∗∗∗p < 0.001, ∗∗∗∗p < 0.0001.

## 3 Results

### 3.1 Reduced adipocyte HS sulfation does not promote diet-induced obesity

**Figure 1:**
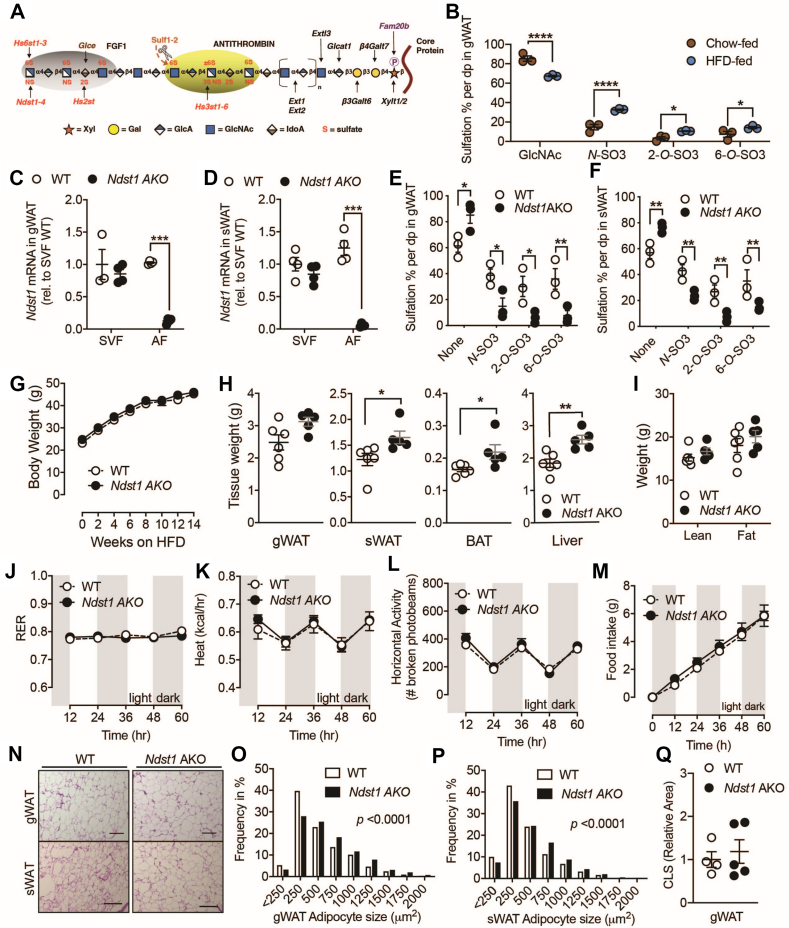
**Reduced sulfation of adipocyte heparan sulfate does not promote obesity**. a, Schematic representation of Heparan Sulfate (HS), including HS biosynthetic enzymes and ligand binding site. **b**, HS *N-, 2-O, and 6-O*-sulfation levels in gWAT of WT mice fed chow or 60 % HFD for 20 weeks. **c**, *Ndst1* mRNA levels in SVF and AF from gWAT. **d**, *Ndst1* mRNA levels in SVF and AF from sWAT. **e**, HS *N-, 2-O, and 6-O*-sulfation levels in AF from gWAT of *Ndst1* AKO and WT mice fed 60 % HFD for 16 weeks. **f**, HS *N-, 2-O, and 6-O-*sulfation levels in AF from sWAT of *Ndst1* AKO and WT mice fed 60 % HFD for 16 weeks **g**, Body weight of *Ndst1* AKO (n = 29) and WT (n = 28) mice fed 60 % HFD. **h**, Tissue weight of *Ndst1* AKO and WT mice fed 60 % HFD for 17 weeks. **i**, DEXA analysis of lean and fat weight of *Ndst1* AKO and WT mice fed 60 % HFD for 17 weeks. **j-m**, Metabolic assessment in metabolic cages of *Ndst1* AKO (n = 5) and WT (n = 6) fed 60 % HFD for 12 weeks. **j**, Respiratory Exchange Ratio (RER). **k**, Energy consumption. **l**, Horizontal activity levels. **m**, Food consumption. **n**, H&E staining of gWAT and sWAT from *Ndst1* AKO and WT mice fed 60 % HFD for 17 weeks. **o-p** quantification of adipocye size in (**o**) gWAT and (**p**) sWAT, **q**, quantification of crown-like structures. Data show mean ± s.e.m., ∗p < 0.05 and ∗∗p < 0.01 vs. WT HFD.

While the complete loss of HS production is not viable in the human population, there is considerable variability in the degree of HS sulfation (Figure 1A) observed between individuals that can range between 10 and 30% [11,19]. When mice are put on a high fat diet (HFD) to induce diet-induced obesity and diabetes the overall sulfation of HS derived from adipose tissue increased 2-fold (Fig. 1B) without significantly altering the overall HS quantity (Supplementary Fig. 1a). *N*-sulfation significantly increase in the HFD mice (Fig. 1B), suggesting a role for adipose tissue HS in metabolic regulation under nutrient stressors. To study the impact of changes in HS composition on obesity and T2D development we inactivated the sulfotransferase, *Ndst1,* in adipocytes (*Ndst1*^*flox/flox*^ *Adipoq-Cre* ^+^ [*Ndst1* AKO]) and put the mice on a HFD (Figure 1A & Supplementary Fig. 1b). *Ndst1* and *Ndst2* are the only *N*-sulfotransferase isoforms expressed in adipocytes, with *Ndst1* being the predominant isoform (Supplementary Fig. 1b). *Ndst1* is considered the primary HS sulfation enzyme for most tissues [20,21], with *Ndst2* having a secondary role in HS elongation [22]. In HFD fed *Ndst1* AKO mice, *Ndst1* expression was reduced in the AF of gonadal white adipose tissue (gWAT) and subcutaneous WAT (sWAT) by 88% and 96%, respectively (Figure 1C–D). No changes in expression were observed in the SVF (Figure 1C–D). Loss of NDST1 expression reduces the degree of HS sulfation thereby compromising their affinity for ligands and their ability to modulate cell signaling among other functions [20,21]. No significant difference was observed in the total gWAT and sWAT HS between HFD-fed WT and *Ndst1* AKO mice, nor was the expression of core HS proteoglycans and *Ndst2* affected by *Ndst1* deletion in adipose tissue (Supplementary Fig. 1c-e). As expected, *Ndst1* deletion resulted in approximately 50% reduction in total N-sulfation in the AF of gWAT and sWAT. Since *N*-sulfation serves as an initiator for downstream sulfotransferases, a concomitant 50–70% reduction in 2-*O* and 6-*O* sulfation was observed while the proportion of unsulfated HS disaccharide units increased (Figure 1E–F). Notably, *Ndst1* inactivation in adipose tissue resulted in similar sulfated HS observed in adipose tissue from chow-fed mice (Fig. 1B) and the residual *N*-sulfation may be attributed to compensatory activity in the absence of NDST1 from the other isoforms (Fig. 1A) [23,24].

*Ndst1* AKO mice on a HFD had similar weight gain as littermate controls (Fig. 1G). There was a modest increase in sWAT, BAT, and liver mass (Fig. 1H). However, the lean and fat weight as measured by DEXA were not significantly altered (Fig. 1I). In conjunction we did not observe any difference in energy substrate utilization and energy expenditure (Figure 1J–K and Supplementary Fig. 1f-g), activity levels (Figure 1l & Supplementary Fig. 1h-i), or food and water intake (Figure 1M and Supplementary Fig. 1j). Histological analysis revealed that the size of adipocytes in the WAT pads from *Ndst1* AKO mice on a HFD for 16-weeks were significantly larger on average compared to WT control (Fig. 1N-p). However, this did not result in changes in the amount of crown-like structures (Fig. 1Q). Plasma adiponectin and leptin levels were also not different between WT and *Ndst1* AKO mice (Supplementary Fig. 1k-l). Chow-fed *Ndst1* AKO mice did not show obvious phenotypes and had normal body and organ weights (Supplementary Fig. 1m-n). Taken together, these data suggest that altered adipocyte HS sulfation composition does not affect the development of diet-induced obesity.

### 3.2 Reduced adipocyte HS sulfation impairs glucose tolerance and insulin sensitivity

**Figure 2:**
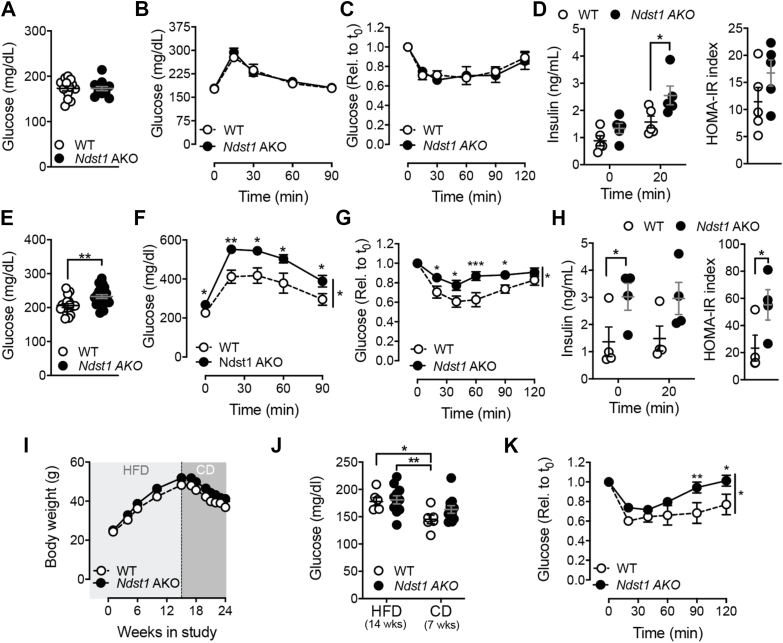
**Reduced sulfation of adipocyte heparan sulfate accelerates insulin resistance.** a, Fasting plasma glucose levels of *Ndst1* AKO and WT mice fed with a CD for 8 weeks **b**, GTT on 24-week-old CD fed *Ndst*1 AKO and WT mice (n = 8–9 per group). **c**, ITT on 25-week-old CD fed *Ndst1* AKO and WT mice (n = 4–5 per group). **d**, Plasma insulin levels of *Ndst1* AKO and WT mice fed CD for 12 weeks pre- and 20 min post-glucose gavage and HOMA-IR was calculated using fasting glucose and insulin levels of 12-week CD fed *Ndst1* AKO and WT mice. **e**, Fasting plasma glucose levels of *Ndst1* AKO and WT mice fed with 60 % HFD for 8 weeks. **f**, GTT on 14-week HFD fed *Ndst1* AKO and WT mice. **g**, ITT on 15-week HFD fed *Ndst1* AKO (n = 12) and WT (n = 6) mice. **h**, Plasma insulin levels of *Ndst1* AKO and WT mice fed HFD for 12 weeks pre and 20 min post glucose gavage and HOMA-IR was calculated using fasting glucose and insulin levels of 12-week HFD fed *Ndst1* AKO and WT mice. **i**, Body weight of *Ndst1* AKO and WT mice fed 60 % HFD for 14 weeks followed by 8 weeks of CD. **f**, Tissue weight of *Ndst1* AKO and WT mice fed 60 % HFD for 16 weeks. **j**, Fasting plasma glucose levels of *Ndst1* AKO (n = 13) and WT (n = 7) mice fed with 60 % HFD for 14 weeks and after 7 weeks of CD. **k**, ITT on 14-week HFD plus 8 weeks of CD fed *Ndst1* AKO (n = 11) and WT (n = 5) mice. Data show mean ± s.e.m., ∗p < 0.05, ∗∗p < 0.01, ∗∗∗p < 0.001, ∗∗∗∗p < 0.0001 vs. WT HFD.

The next objective was to evaluate if decreased HS sulfation on adipocytes affects glucose homeostasis by testing glucose tolerance and insulin sensitivity in chow and HFD-fed *Ndst1* AKO mice. Glucose levels were unaffected in *Ndst1* AKO mice on chow diet (Figure 2A), as was glucose tolerance (Fig. 2B) and insulin sensitivity (Fig. 2C). However, when measuring insulin levels before and after a glucose bolus, a significant increase in insulin could be observed in *Ndst1* AKO mice compared to WT littermates (Fig. 2D). Using the HOMA-IR index as a measure of insulin resistance, the *Ndst1* AKO mice showed a trend towards increased HOMA-IR even on a chow diet (Fig. 2D).

Challenging *Ndst1* AKO mice with HFD increased fasting glucose levels (Fig. 2E) and decreased glucose tolerance (Fig. 2F) and insulin sensitivity (Fig. 2G). Reduced glucose tolerance and insulin sensitivity was already observed after 6-weeks of HFD feeding (Supplementary Fig. 2a-b). As expected, HFD-fed *Ndst1* AKO mice had increased insulin secretion in the basal state, and a trend toward higher glucose-stimulated insulin secretion after 12-weeks HFD compared to WT controls (Fig. 2H), resulting in a significantly increased HOMA-IR index (Fig. 2H). To test if *Ndst1* AKO mice had reached a more advanced and less reversible stage of T2D, WT and *Ndst1* AKO mice with comparable fasting glucose levels after 14-weeks of HFD feeding were switched to a low-fat chow diet (Fig. 2I) [25,26]. Both WT and *Ndst1* AKO mice lost an equal amount of body weight after 8 weeks of chow diet (Fig. 2I). The diet switch significantly reduced fasting glucose levels in WT mice (Fig. 2J). In contrast, fasting glucose levels remained unchanged (Fig. 2J) and insulin resistance remained prominent (Fig. 2K) in *Ndst1* AKO mice after 8 weeks on a low-fat chow diet. Together the data support that reduced adipocyte HS sulfation negatively affects DIO-associated glucose tolerance and metabolic flexibility during weight loss.

### 3.3 Total loss of adipocyte heparan sulfate affects glucose homeostasis upon high fat diet feeding

To confirm the robustness of our finding we also examine the impact of total loss of adipocyte HS chain generation on diet-induced obesity (DIO) and type-2 diabetes susceptibility by genetically targeting Exostosin 1 (*EXT1*). EXT1 is an essential enzyme for HS chain elongation after generation of the tetrasaccharide linker region at the protein core (Fig. 1A). Its inactivation prevents HS production, making systemic EXT1 deletion not viable [27]. To circumvent this limitation as well as the requirement for HS in pre-adipocyte differentiation, we utilized an adiponectin-driven Cre that is only expressed in mature adipocytes in combination with *Ext1*-floxed mice. *Ext1*^*flox/flox*^ *Adipoq-Cre*^+^ (referred to as *Ext1* AKO) mice were viable and did not show obvious phenotypes on chow diet (Supplementary Fig. 3a). *Ext1* AKO mice presented with a 60% decrease in *Ext1* expression in the adipocyte fraction (AF) and not in the stromal vascular cell fraction (SVF) (Supplementary Fig. 3b). It is likely that the remaining expression results from a contamination of stromal cells that remained in the adipocyte fraction as we were unable to isolate any detectable HS from the adipocyte fraction.

**Figure 3:**
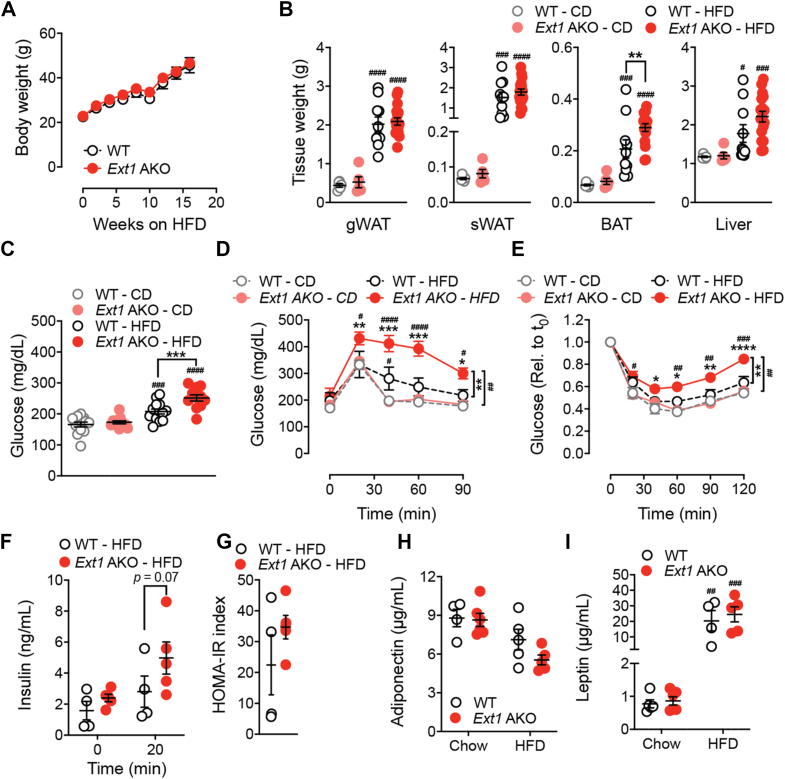
**Loss of adipocyte heparan sulfate accelerates type-2 diabetes development.** a, Body weight of *Ext1* AKO (n = 17) and WT (n = 11) mice fed with CD or 60 % HFD. **b**, Tissue weight of *Ext1* AKO and WT mice fed with 60 % HFD for 16 weeks. **c**, Fasting plasma glucose levels of *Ext1* AKO and WT mice fed with CD or 60 % HFD for 8 weeks **d**, GTT on 14-week CD or HFD fed *Ext1* AKO (n = 6) and WT (n = 6–8) mice. **e**, ITT on 15-week CD or HFD fed *Ext1* AKO (n = 5–12) and WT (n = 6–14) mice. **f**, Plasma insulin levels of *Ext1* AKO and WT mice fed HFD for 12 weeks pre and 20 min post glucose gavage. **g**, HOMA-IR was calculated using fasting glucose and insulin levels of 12-week HFD fed *Ext1* AKO and WT mice. **h**, Fasting plasma adiponectin levels of *Ext1* AKO and WT mice fed with CD or 60 % HFD for 12 weeks. **i**, Fasting plasma leptin levels of *Ext1* AKO and WT mice fed with CD or 60 % HFD for 12 weeks. Data show mean ± s.e.m., ∗p < 0.05, ∗∗p < 0.01, ∗∗∗p < 0.001, ∗∗∗∗p < 0.0001 vs. WT HFD; #p < 0.05, ##p < 0.01, ###p < 0.001, ####p < 0.0001 vs. CD of same genotype.

To evaluate the impact of adipocyte HS on diet-induced obesity and T2D, we placed 8-week-old male *Ext1* AKO and WT (wildtype; *Ext1*^*flox/flox*^ *Adipoq-Cre*^-^) mice on a high fat diet (HFD; 60% calories in fat) for 15 weeks. Similar to *Ndst1* AKO mice the *Ext1* AKO mice did not exhibit differences in total weight gain upon HFD (Figure 3A) except for a moderate increase in BAT (Fig. 3B). However, HFD-fed *Ext1* AKO mice presented with elevated fasting plasma glucose levels compared to WT controls (Fig. 3C). This was accompanied by decreased glucose sensitivity (Fig. 3D) and increased insulin resistance in *Ext1* AKO mice as measured by glucose and insulin tolerance tests, respectively, after 15-weeks of HFD (Fig. 3E). The metabolic phenotype was also observed at early stages of HFD-feeding (7–8 weeks on HFD, Supplementary Fig. 3c-d). The trend towards augmented glucose-induced insulin release supports increased insulin need in *Ext1* AKO mice compared to 10.13039/100010269WT littermate controls when placed on a HFD, despite the HOMA-IR index not being different (Figure 3F–G); a phenotype that was independent of plasma adiponectin and leptin levels (Figure 3H–I). The predisposition of *Ext1* AKO mice to develop diabetes was HFD-depended as no differences in organ weight, fasting plasma glucose, glucose tolerance, and insulin sensitivity were observed between WT and *Ext1* AKO mice maintained on a chow diet (Figure 3B–C). These data establish that adipocyte-derived HS and HS sulfation influence glucose and insulin homeostasis during nutrient excess but do not affect weight gain.

### 3.4 *Ndst1* AKO mice are predisposed to diet-induced hepatic steatosis and insulin resistance

**Figure 4:**
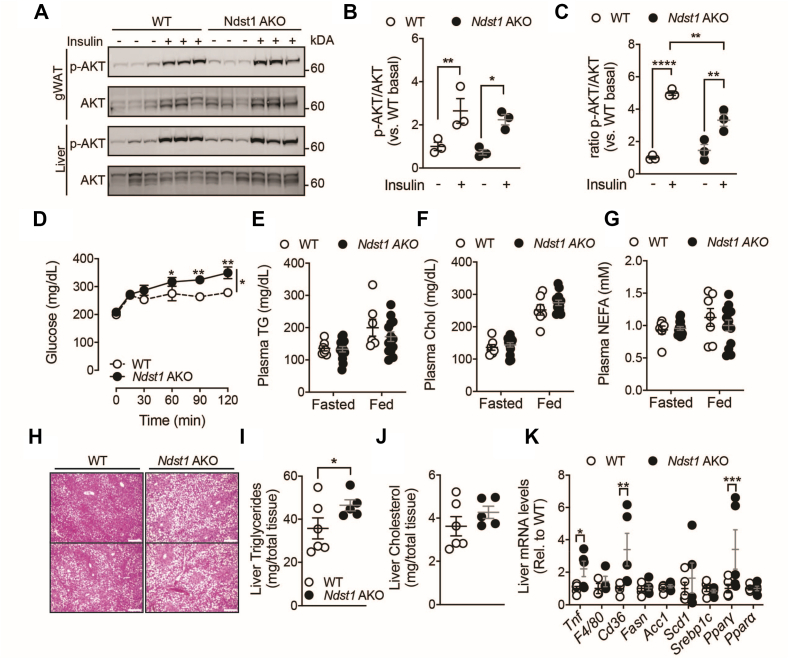
**Increased Liver steatosis and insulin resistance *Ndst1* AKO mice.** a, Western blot analysis and quantification of AKT and phospho-AKT (p-AKT) in gWAT and liver of 16-week HFD fed WT and *Ndst1* AKO mice 20 min after a single dose of insulin (1 U/kg). **b**, Densitometric analysis of Western blot bands in gWAT. **c**, Densitometric analysis of Western blot bands in liver **d**, PTT after 10 weeks of HFD in *Ndst1* AKO (n = 14) and WT (n = 12). **e-g**, Plasma lipid parameters in fed *ad libitum* and overnight fasted state in 12-week HFD-fed WT and *Ndst1* AKO mice; **e**, triglyceride levels, **f**, cholesterol, and **g**, NEFA. **h**, Representative H&E images of livers of WT and *Ndst1* AKO mice harvested after 12 weeks of HFD (Scale bar = 250 μm). **i**, Liver triglyceride levels of 16-week HFD fed WT and *Ndst1* AKO mice. **j**, Liver cholesterol levels of 16-week HFD fed WT and *Ndst1* AKO mice. **k**, mRNA analysis of liver of 12-week HFD fed WT and Ndst1 AKO mice. Data show mean ± s.e.m., ∗p < 0.05, ∗∗p < 0.01, ∗∗∗p < 0.001, ∗∗∗∗p < 0.0001 vs. WT HFD.

We next evaluate if adipocyte HS is required for normal insulin signaling in the more human relevant *Ndst1* AKO mouse model. We injected fasted HFD-fed *Ndst1* AKO and WT mice with insulin (1U/kg) and harvested gWAT and liver 20 min after injection. Insulin induced the phosphorylation of protein kinase B (p-AKT), a known effector molecule of the insulin signaling pathway. Phosphorylation was similarly induced in gWAT from both WT and *Ndst1* AKO mice (Figure 4A–B) indicating that the insulin signaling capacity of AT was not compromised by the absence of properly sulfated HS. However, a trend towards decreased p-AKT was observed in the livers of *Ndst1* AKO mice (Figure 4A,C), suggesting increase liver insulin resistance. This was confirmed by a pyruvate tolerance test, showing significantly increased gluconeogenesis deregulation in *Ndst1* AKO mice compared to WT controls (Fig. 4D); a phenotype that was not observed in chow fed mice (Supplementary Fig. 4a). The adipose *Ndst1* deletion did not affect circulating triglyceride, cholesterol, or non-esterified fatty acid (NEFA) levels, or plasma lipoprotein distribution (Figure 4E–G; Supplementary Fig. 4b-h). However, *Ndst1* AKO mice presented with a marked increase in hepatic steatosis, as revealed by histological analyses (Fig. 4H). Livers from *Ndst1* AKO mice had elevated triglyceride levels, suggesting advanced fatty liver disease (Figure 4I–J). Consistent with this, hepatic expression of *Tnf*, *Cd36, and Ppary* was increased in *Ndst1* AKO mice (Fig. 4K). Collectively, *Ndst1* AKO mice show signs of increased hepatic inflammation, insulin resistance, and liver lipid accumulation in a DIO model.

### 3.5 FGF1 binding and signaling is reduced in HS deficient adipocytes

**Figure 5:**
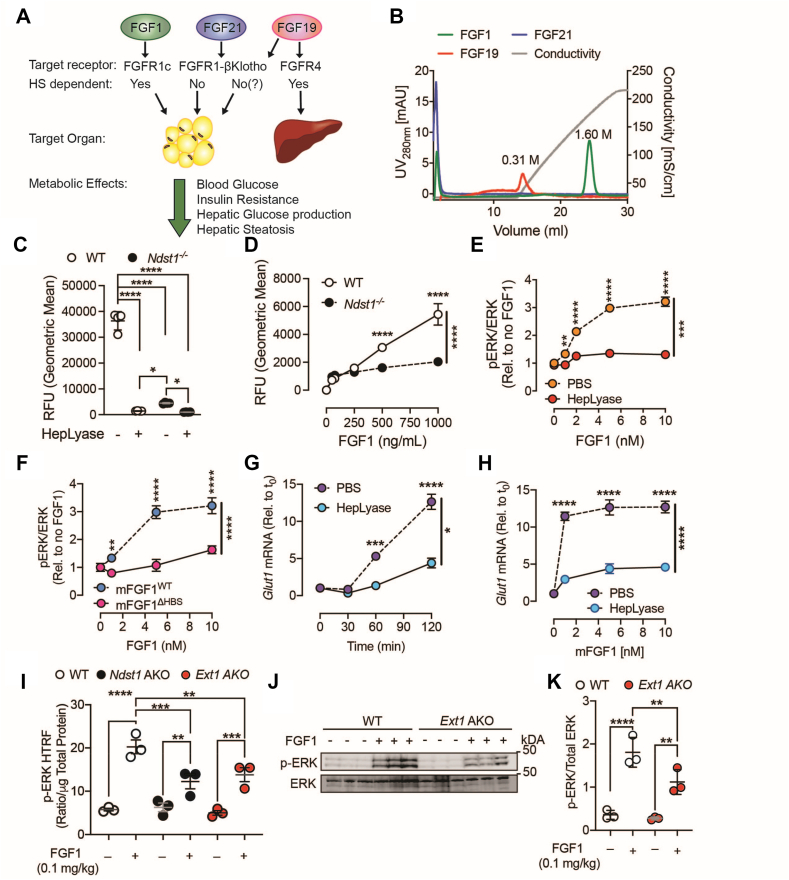
**FGF1 signaling in adipocytes is co-regulated by cell surface heparan sulfate.** a, Schematic overview of metabolic actions and HS dependency of endocrine FGF's. **b**, Binding of recombinant FGFs to a heparin Sepharose column. The numbers above the peaks signify the NaCl concentration in molar required for elution of the heparin column calculated from the conductivity. **c**, Binding of 10 μg/ml mFGF1^WT^ to WT and *Ndst1*^*−/−*^ MEF pre-adipocytes without and with HepLyase pre-treatment (4 mU/mL for 30 min). **d**, Dose curve of rFGF1 binding on WT and *Ndst1*^−/−^ MEF-derived adipocytes (n = 3). **e-f**, Quantification of phospho-ERK (p-ERK) activation using homogenous time-resolved fluorescence resonance energy transfer (HTRF) after 5 min of murine FGF1 administration in 3T3-L1 derived adipocytes without and with HepLyase pre-treatment (0.5 U/mL for 30 min; n = 3) or treated with wildtype murine FGF1 (mFGF1^WT^) and murine FGF1 lacking the HS binding sequence (mFGF1^ΔHBS^) (n = 4). **g-h**, Time course analysis (**g**) and dose curve (**h**) of *Glut1*gene expression after treatment with FGF1 in 3T3-L1 derived adipocytes without and with HepLyase pre-treatment (0.5 U/mL for 30 min; n = 4). **i**, HTRF analysis of mFGF1^WT^-induced ERK phosphorylation in gWAT of WT, *Ndst1* AKO, and *Ext1* AKO mice. **j-k**, (**j**) Western blot analysis and (**k**) quantification of mFGF1^WT^-induced ERK phosphorylation in gWAT of WT and *Ext1* AKO mice. Data show mean ± s.e.m., ∗p < 0.05, ∗∗p < 0.01, ∗∗∗p < 0.001, ∗∗∗∗p < 0.0001.

Several studies show that FGF1 produced by adipocytes acts a potent paracrine insulin sensitizer on AT(Figure 5A) [[28], [29], [30], [31]]. Genetic inactivation of FGF1 expression was associated with impaired glucose tolerance, insulin resistance, increased gluconeogenesis, and hepatic steatosis in DIO models (Fig. 5A). FGF1 expression and release in adipocytes is regulated by nutrient sensing via peroxisome proliferator-activated receptor γ (PPARγ) activation [32] and mechanosensing of adipocyte expansion via Piezo1, respectively [33]. In mature adipocytes FGF1 exerts regulatory control over plasma glucose levels by promoting glucose uptake in adipocytes and inhibiting hepatic gluconeogenesis [34]. FGF1 exhibits strong affinity for HS, distinguishing it from other endocrine FGFs such as FGF15/19 and FGF21. The high affinity for HS creates a local pooling of endogenous FGF1 in the glycocalyx and ECM that surrounds cells, including adipocytes. The HS chain(s) acts as a bridge to approximate FGF1 and FGFR1. The length of the HS chains and their sulfation pattern is relevant for binding FGF1 and is crucial for formation of a paracrine 2:2:2 or 2:2:1 dimer FGF1-FGFR1-HS complex, which facilitates trans-phosphorylation and downstream signaling. Given the requirement for HSPG as co-receptors for FGF1 signaling and the overlapping phenotypes, we hypothesized that paracrine FGF1 signaling is disrupted in the *Ndst1* AKO mice (Fig. 5A). To illustrate this, we compared their heparin binding as a proxy for HS binding. As expected, FGF21 did not bind heparin and FGF19 had modest HS binding and required a 0.31 mM salt concentration to eluate from the column (Fig. 5B). In contrast, FGF1 bound strongly and required 1.6 mM salt to elute from the heparin column (Fig. 5B).

To explore this notion, we initially characterized the consequences of compromising HS on the metabolic effects of exogenous FGF1. In line with previous observations, adipocytes derived from *Ndst1*^*−/−*^ mouse embryonic fibroblasts (MEFs) showed decreased recombinant FGF1 (mFGF1^WT^) binding, comparable to WT MEFs pre-treated with heparan sulfate lyases I, II, III (r) which remove HS presented on the cell surface (Fig. 5C). The HepLyase treatment did not significantly decrease binding of mFGF1^WT^ in *Ndst1*^*−/−*^ pre-adipocytes (Fig. 5C). The reduction in FGF1 binding in the *Ndst1*^−/−^ adipocytes was not due shortening of the HS chain length, in fact the average HS chain length was larger in the *Ndst1*^−/−^ adipocytes compared to the WT controls (Supplementary Fig. 5a). mFGF1^WT^ showed dose-dependent binding to WT adipocytes, which was markedly reduced in *Ndst1*^*−/−*^ pre-adipocytes (Fig. 5D). In 3T3-L1-derived adipocytes, pretreatment with HepLyases attenuated FGF1-induced signaling, as measured by ERK phosphorylation (Fig. 5E). Similar results were obtained in MEF-derived adipocytes (Supplementary Fig. 5b-d). Treatment of 3T3-L1 derived adipocytes with murine FGF1 lacking the HS binding sequence (mFGF1^ΔHBS^) [35] was also unable to induce FGF1-induced ERK phosphorylation to the same extend as murine wildtype FGF1 (mFGF1^WT^) (Fig. 5F). Previous work identified that FGF1 induces expression of the glucose transporter GLUT1 in a dose and time dependent manner [36]. Similarly treatment of 3T3-L1 derived adipocytes with FGF1 induced *Glut1* mRNA levels by 12.7-fold after 120 min of stimulation (Figure 5G–H). Removal of cell surface HS via HepLyase treatment significantly blunted the response by 66% and resulted in only a 4.3 increase in *Glut1* mRNA levels in a time and dose dependent fashion (Figure 5G–H). Building upon these findings *in vitro*, we investigated *in vivo* mFGF1^WT^-induced ERK phosphorylation in gWAT of WT, *Ndst1* AKO, and *Ext1* AKO mice. Intraperitoneal injection of mFGF1^WT^ (0.1 mg/kg) fasted body weight dose for 20 min yielded a significant increase in ERK phosphorylation, but the response was significantly attenuated in gWAT isolated from *Ndst1* AKO and *Ext1* AKO mice (Figure 5I–K). Taken together, these results suggest that proper HS sulfation and cell surface presentation are essential for robust metabolic FGF1 signaling in adipocytes.

### 3.6 FGF1-induced glucose lowering is impaired in *Ndst1* AKO mice

**Figure 6:**
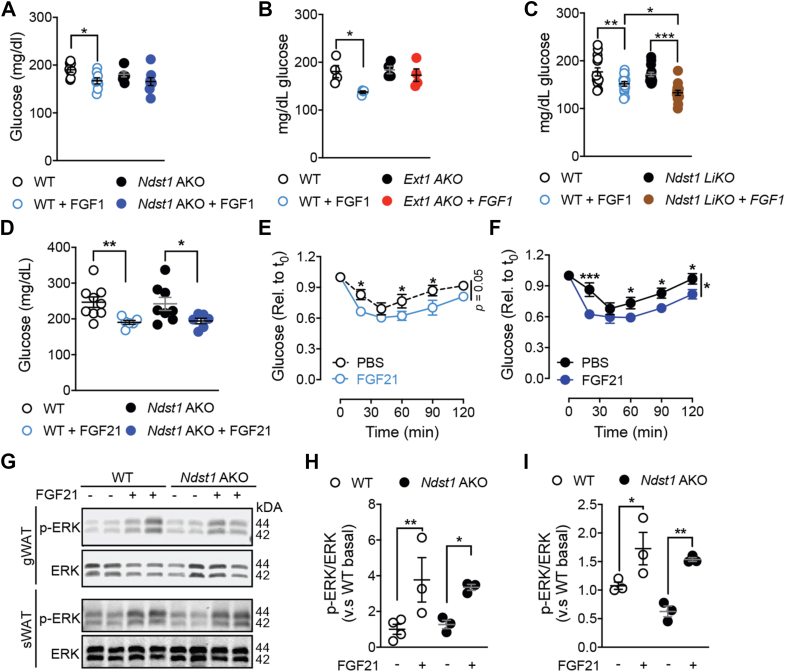
**Recombinant FGF1 treatment requires adipocyte HS for acute glucose lowering.a**, Glucose levels 12 h after a single i.p. injection of rFGF1 (0.5 mg/kg) or PBS in WT and *Ndst1* AKO mice fed HFD for 12 weeks **b**, Glucose levels 12hrs. after a single i.p. injection of rFGF1 (0.5 mg/kg) or PBS in WT and *Ext1* AKO mice fed HFD for 12 weeks. **c**, Glucose levels 12 h after a single i.p. injection of rFGF1 (0.5 mg/kg) or PBS in WT and *Ndst1* LiKO mice fed HFD for 12 weeks **d**, Glucose levels 12 h after a single i.p. injection of rFGF21 (0.5 mg/kg) or PBS in WT and *Ndst1* AKO mice fed HFD for 12 weeks. **e**, ITT after a single (0.5 mg/kg) rFGF21 or PBS injection into 14-week HFD fed WT mice (n = 8). **f**, ITT after a single (0.5 mg/kg) rFGF21 or PBS injection into 14-week HFD fed *Ndst1* AKO mice (n = 6). **g**, Western blot analysis of ERK and p-ERK in gWAT and sWAT of WT and *Ndst1* AKO mice fed a HFD for 15-weeks 20 min after a single dose of 0.5 mg/kg rFGF21. **h**, Densitometric analysis of Western blot bands in gWAT. **i**, Densitometric analysis of Western blot bands in sWAT. Data show mean ± s.e.m., ∗p < 0.05, ∗∗p < 0.01, ∗∗∗p < 0.001.

Previous studies showed that acute administration of recombinant FGF1 (rFGF1) reduced glucose levels in obese, insulin resistant mice within 12-hrs (Figure 6A–C) [30]. To explore the dependency of this effect on HS sulfation, HFD-fed WT and *Ndst1* AKO mice with matched basal glucose levels were given a single intra-peritoneal (i.p.) injection of 0.5 mg/kg recombinant FGF1 (rFGF1). Notably, rFGF1 failed to lower blood glucose levels in *Ndst1* AKO mice (Fig. 6A). Similarly, the blood glucose levels in HFD-fed *Ext1* AKO mice were not affected by a single rFGF1 injection (Fig. 6B).

Liver expresses abundant amounts of highly sulfated HSPGs, which are important in modulating lipoprotein homeostasis [37]. We hypothesized that liver HS can be a sink for i.p. injected rFGF1 and reduce AT targeting. To evaluate this notion, we placed *Ndst1* liver-specific knock-out mice (*Ndst1*^f/f^ *Alb-*Cre^+;^ *Ndst1* LiKO) on a HFD. Loss of liver *Ndst1* did not affect weight gain, basal glucose levels, glucose tolerance, insulin resistance, or pyruvate tolerance, but did result in increased plasma triglyceride-rich lipoprotein levels compared to WT controls at 16-weeks of HFD feeding (Supplementary Fig. 6a-j). However, reduced HS sulfation in the HFD-fed *Ndst1* LiKO mice led to a more pronounced glucose lowering effect compared to WT mice 12-hrs post a 5 mg/kg rFGF1 injection, in line with our hypothesis (Fig. 6C).

In contrast to the paracrine FGF1, the endocrine FGF21 signals independently of HS [38,39]. Therefore we hypothesized that under-sulfated adipose HS should not influence the metabolic actions of FGF21. Supporting this notion rFGF21 injections had similar insulin sensitizing effects in HFD-fed WT and *Ndst1* AKO (Figure 6D–F). Moreover, MAP-kinase signaling following rFGF21 injection showed that ERK phosphorylation in gWAT and sWAT was not changed in *Ndst1* AKO mice (Figure 6G–I and Supplementary Fig. 6k-l). Our data illustrate that adequate adipocyte HS sulfation is crucial for acute FGF1 glucose lowering, but not for the HS-independent endocrine FGF21 signaling.

### 3.7 Chronic rFGF1 treatment requires uncompromised adipocyte heparan sulfate

**Figure 7:**
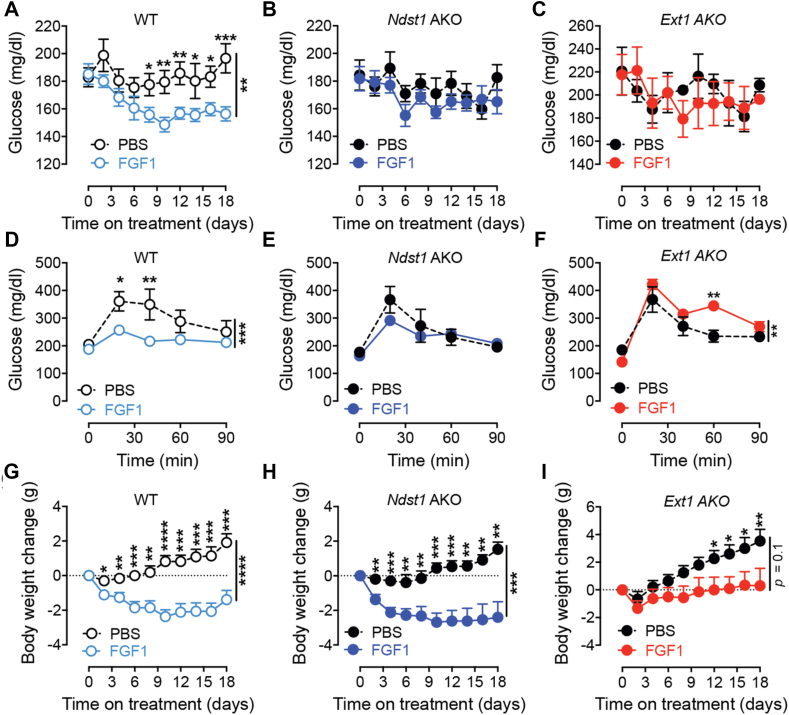
**Long term rFGF1 treatment does not lower plasma glucose levels when adipocyte-derived HS is compromised.** a, Glucose levels measured during an 18-day bi-daily i.p injection of rFGF1 (0.5 mg/kg) or PBS in WT mice after 16 weeks of HFD (n = 9–10 per group). **b,** Glucose levels measured during an 18-day bi-daily i.p injection of rFGF1 (0.5 mg/kg) or PBS in *Ndst1* AKO mice after 16 weeks of HFD (n = 9 per group). **c,** Glucose levels measured during an 18-day bi-daily i.p injection of rFGF1 (0.5 mg/kg) or PBS in *Ext1* AKO mice after 16 weeks of HFD (n = 3 per group). **d**, GTT measured after 18-days of bi-daily i.p injection of rFGF1 (0.5 mg/kg) or PBS in WT mice after 16 weeks of HFD (n = 5 per group). **e**, GTT measured after 18-days of bi-daily i.p injection of rFGF1 (0.5 mg/kg) or PBS in *Ndst1* AKO mice after 16 weeks of HFD (n = 4–5 per group). **f**, GTT measured after 18-days of bi-daily i.p injection of rFGF1 (0.5 mg/kg) or PBS in *Ndst1* AKO mice after 16 weeks of HFD (n = 3 per group). **g**, Body weight change of WT mice during rFGF1 treatment (n = 9–10 per group). **h**, Body weight change of *Ndst1* AKO mice during rFGF1 treatment (n = 9 per group). **i**, Body weight change of *Ext1* AKO mice during rFGF1 treatment (n = 3 per group). Data show mean ± s.e.m., ∗p < 0.05, ∗∗p < 0.01, ∗∗∗p < 0.001, ∗∗∗∗p < 0.0001.

To test if adipocyte HS is required for glucose lowering induced by prolonged FGF1 administration we injected every other day 0.5 mg/kg rFGF1 or excipient control (PBS) intraperitoneal in HFD-fed WT, *Ndst1* and *Ext1* AKO mice for 18 days (Supplementary Fig. 7a). The mice were fed a HFD for 12 weeks before onset of the treatment, which was maintained during the treatment period. rFGF1 treatment significantly reduced fasting glucose levels in WT mice seven days into the treatment compared to PBS injected controls (Figure 7A). In contrast, rFGF1 injections did not significantly improve glucose levels throughout the 18-day treatment period in *Ndst1* AKO and *Ext1* AKO mice (Figure 7B–C). The rFGF1 treatment improved glucose tolerance in WT animals (Fig. 7D), however no significant improvements were observed in *Ndst1* AKO mice (Fig. 7E), and *Ext1* AKO mice were less glucose tolerant at the end of the treatment (Fig. 7F). A transient reduction in body weight was seen in WT and *Ndst1* AKO mice treated with rFGF1; likely the consequence of a centrally-mediated reduction in food intake that is expected to be independent of adipocyte HS status [40] (Figure 7G,H). However, despite the weight loss, *Ndst1* AKOs mice failed to show improvements in glucose homeostasis. Interestingly, *Ext1* AKO mice appear protected from FGF1-induced weight loss (Fig. 7I).

While two FGF1 transcripts are expressed in AT, only *Fgf1a* is feed/fasting regulated and increased upon HFD [29]. In WT mice, *Fgf1a* expression was reduced by chronic rFGF1 treatment, suggestive of a compensatory response. Interestingly, while there was a trend towards lower basal expression of *Fgf1a* in *Ndst1* AKO, this was insensitive to rFGF1 treatment (Supplementary Fig. 7b). Expression of *Fgf1b* and *Fgfr1c,* the alternative adipose transcript and relevant FGF receptor, respectively, were not affected by chronic rFGF1 treatment or the loss of *Ndst1* in adipocytes (Supplementary Fig. 7c-d). Together, our data support that the degree of adipocyte HS sulfation is crucial for the glucose lowering action of FGF1. Thus, animals lacking either *Ndst1* or *Ext1* in adipocytes appear predisposed to develop peripheral FGF1 resistance in response to a dietary challenge.

### 3.8 *Ndst1* AKO mice phenocopy the adipose-specific loss of FGFR1

**Figure 8:**
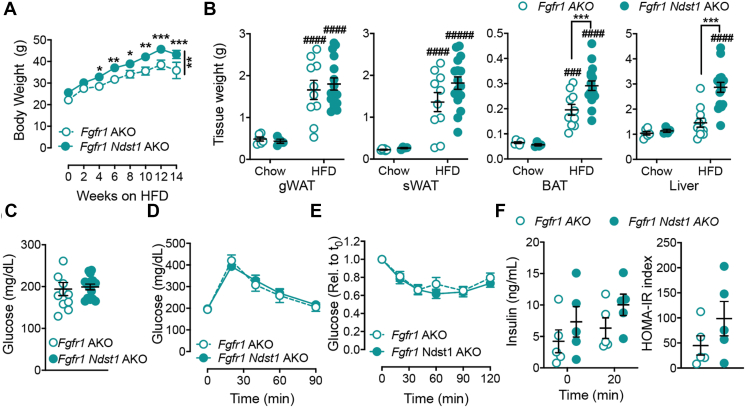
**HS on adipocytes do not affect glucose metabolism in the absence of FGFR1.** a, Body weight of *Fgfr1* AKO (n = 11) and *Fgfr1 Ndst1* AKO (n = 16) mice fed 60 % HFD. **b**, Tissue weight of *Fgfr1* AKO and *Fgfr1 Ndst1* AKO mice fed 60 % HFD for 16 weeks or CD controls. **c**, Fasting plasma glucose levels of *Ndst1* AKO and WT mice fed with a CD for 8 weeks **d**, GTT of 8-week HFD fed *Fgfr1* AKO (n = 11) and *Fgfr1 Ndst1* AKO (n = 16) mice. **e**, ITT of 9-weeks HFD fed *Fgfr1* AKO (n = 10) and *Fgfr1 Ndst1* AKO (n = 14) mice. **f**, Plasma insulin levels of *Fgfr1* AKO and *Fgfr1 Ndst1* AKO mice fed HFD for 12 weeks pre and 20 min post glucose gavage and HOMA-IR was calculated using fasting glucose and insulin levels of 12-week CD fed *Ndst1* AKO and WT mice. Data show mean ± s.e.m., ∗p < 0.05, ∗∗p < 0.01, ∗∗∗p < 0.001 vs. WT HFD; ###p < 0.001, ####p < 0.0001 vs. CD of same genotype.

Our findings associate the metabolic phenotype of *Ndst1* AKO mice with a reduction in FGF1 signaling in adipocytes. To explore this notion, we bred adipocyte-specific *Ndst1* AKO mice on the adipose-specific *Fgfr1* knockout background (*Fgfr1*^*flox/flox*^ Adipoq-Cre^+^; *Fgfr1 AKO*) to generate *Fgfr1 Ndst1* AKO (*Fgfr1*^*flox/flox*^*Ndst1*^*flox/flox*^ Adipoq-Cre^+^) mice. On a chow diet the *Fgfr1* AKO and *Fgfr1 Ndst1* AKO showed no difference in weight gain, glucose levels, glucose tolerance and insulin resistance (Supplementary Fig. 8a-d). However, loss of *Ndst1* in combination with *Fgfr1* deletion resulted in increased weight gain on HFD compared to *Fgfr1* AKO mice (Figure 8A). The weight gain is mostly accounted for by increased liver weight which was apparent after 16 weeks of HFD (Fig. 8C). Also, BAT mass increased in *Fgfr1 Ndst1* AKO mice compared to *Fgfr* AKO, while sWAT and gWAT mass remained unchanged (Fig. 8B).

Despite the increase in body weight, no differences in plasma glucose levels (Fig. 8C), glucose tolerance (Fig. 8D), or insulin sensitivity were observed between *Fgfr1 Ndst1* AKO and *Fgfr1* AKO mice (Fig. 8E). Similarly, glucose-induced insulin secretion and HOMA-IR values were not different (Fig. 8F). Longer HFD-feeding (15-weeks) did not significantly alter this phenotype (Supplementary Fig. 8e-f). Hence, loss of *Ndst1* in addition to *Fgfr1* does not compound the dysregulation of glucose homeostasis and supports the concept that adipose HS is a critical co-regulator of metabolic FGF1 signaling in adipocytes.

## 4 Discussion

Here we demonstrate that HS presented by mature adipocytes co-regulate systemic glucose homeostasis under high fat diet feeding by promoting metabolic FGF1 signaling. The effects were independent of weight gain and adiposity. The lack of an effect on body weight and food intake implies that HS produced by mature adipocytes does not directly impact adipocyte expansion. This observation contrasts *Ext1*^*flox/+*^ *Ap2-Cre* ^+^ mice [13], where heterozygous *Ext1* deletion reduces weight gain and adiposity upon HFD administration. Unlike *Adipoq-Cre* deletion, the *Ap2-Cre*-system is not as specific and inactivates floxed-target genes in multiple tissues, including skeletal muscle, macrophages, and adipocyte progenitor cells [41,42]. Given the importance of HS in pre-adipocyte differentiation, it is more than conceivable that the reduced weight gain is a result of impaired adipogenesis and lipid storage due to impaired HS chain elongation [13,14]. Targeting HS in adipocyte progenitor cells might also explain why homozygous Ext1 deletion using the *Ap2-Cre*-system (*Ext1*^*flox/flox*^ *Ap2-Cre*^+^) resulted in embryonic lethality, which was not observed in *Ext1*^*flox/flox*^ *Adipoq-Cre* ^+^ mice. *Ap2*-Cre mediated *Ext1* deletion in macrophages is likely not contributing to the reduced weight in *Ext1*^*flox/+*^ *Ap2-Cre* ^+^ mice as we have shown before that decreased HS sulfation in macrophages promotes obesity and insulin resistance [43].

Compromising HS in adipocytes in DIO mice strongly associated with enlarged BAT and livers with an increase in hepatic lipid accumulation and inflammation, accompanied by impaired gluconeogenesis. Impaired adipose HS may modulate systemic glucose tolerance by disrupting HSPG-dependent growth factor signaling, such as FGF1–FGFR1 interactions, thereby altering adipose insulin sensitivity, lipid handling, and whole-body glucose homeostasis. However, in the absence of FGFR1 in adipocytes, additional inactivation of *Ndst1* still results in enlarged BAT, but not WAT, accompanied by liver enlargement. This suggests that when adipose tissue metabolic FGF1 signaling is lost, HS plays a critical role in maintaining BAT function, which may explain the persistent liver phenotype observed in the Fgfr1 Ndst1 AKO mice. Future studies will be required to directly assess BAT function under cold activation conditions to clarify these mechanisms. BAT and also adipose tissue HSPGs, particularly COL18A, are important for promoting triglyceride-rich lipoprotein lipolysis via lipoprotein lipase and remnant uptake in the liver [44]. Compromising peripheral LPL activity should increase plasma triglyceride levels and result in increased dietary lipid flux to the liver. However, *Ndst1* AKO mice have normal plasma lipids levels and an unaltered lipoprotein distribution profile, which makes these pathways unlikely responsible for the liver phenotype. It also implies that the COL18A (present in the adipose tissue ECM) mediating translocation of LPL from adipocytes to GPIHBP1 on endothelium is produced by endothelium or other surrounding cells and not adipocytes.

The increased hepatic steatosis in *Ndst1* AKO mice is most likely a result of FFA spillover from adipose tissue. FGFR1-FGF1 signaling promotes inhibition of hormone-sensitive lipase activity in adipocytes during periods of nutrient excess [30,34,36]. In the event of insulin resistance, the anti-lipolytic activity of FGF1 holds increasing importance as an alternative method of glucose and lipid homeostasis. The reduction of basal *Fgf1* expression in gWAT of *Ndst1* AKO mice suggests a reduced efficacy and dependence on FGFR1-FGF1 signaling arising from reduced HS sulfation. We propose a model in which secreted HSPGs sequester FGF1 near its site of release, thereby restricting its availability and enforcing paracrine signaling [46,47]. In contrast, cell surface–anchored HSPGs such as syndecans and glypicans are the most likely facilitators of FGFR1–FGF1 complex formation due to their proximity to the receptor. Together, this suggests a dual regulatory role where secreted HSPGs shape the local distribution of FGF1, while membrane-tethered HSPGs enable its productive signaling. The absence of HS-dependent metabolic effects in *Fgfr1* AKO mouse models further reinforces FGFR1 as the key receptor HS mediates in FGF1-based metabolic signaling. The combination of poor FGFR1-FGF1 signaling with obesity-induced insulin resistance results in worse lipolytic regulation in the adipose tissue of *Ndst1* AKO mice and leads to the observed increased hepatic steatosis [32,45] and hyperglycemia. These observations implicate HS involvement on adipocyte as a co-regulator of metabolic FGF1-FGFR1 signaling.

The properties of FGF1 have garnered therapeutic interest as a potential endocrine agent [30,[48], [49], [50], [51]]. Peripheral injection of FGF1 in diabetic HFD-fed mice and *ob/ob* mice restored normoglycemia, improved insulin sensitivity, reduced hepatic steatosis and inflammation, independent of weight loss, and without inducing hypoglycemia [30]. These effects rely on FGFR1 expression in adipocytes and are uncoupled from the mitogenic activity of FGF1 [30,35]. Notably, FGF1 injection also exerts effects on the liver (reversing MAFLD) [48], pancreas (enhancing β-cell function) [49], skeletal muscle (promoting GLUT4 expression) [51], cardiomyocytes (reduces oxidation) [50], kidney (reduced inflammation) [52], and brain (anorexigenic) [30,40,49,[53], [54], [55]]. Studies identified that injection of recombinant FGF1 lacking lysine residues in the HS-binding site (FGF1^ΔHBS^) lowers glucose levels in *db/db* mice without improving insulin levels and sensitivity [30,35,50,52]. This finding suggests that the mitogenic and the metabolic activity of FGF1 can be uncoupled by modulating HS. However, the interpretation is complicated by the pleiotropic multi-organ effects of FGF1 and the high dose that is administered to animals. Notably, intravenous FGF1^ΔHBS^ injections into mice resulted in a sustained and severe anorexigenic effect (>25% body weight reduction; ∼50% food intake reduction in mice and 100% in non-human primates) [50,56], a confounding factor that was not observed with wildtype rFGF1 administration [30]. The lack of HS binding also will alter FGF1 biodistribution likely increasing FGF1^ΔHBS^ partitioning to the brain, where HS is less abundant. Notably, FGF1^ΔHBS^ metabolic benefits, such as glucose lowering, were no longer observed when weight loss was not achieved as in *Ampk*-deficient mice [40,50,53,55]. Our results, show that mice with deficient adipocyte HS lack the glucose-lowering response of rFGF1 administration and suggest that the interaction with adipocyte HS is crucial to mediate FGF1 effects on adipose tissue plasticity *in vivo*. The weight loss associated with rFGF1 administration was not altered in mice with compromised adipocyte HS, suggesting that central effects of FGF1 administration were not impacted. However, *Fgf1*^−/−^ mice on HFD do not gain more weight compared to WT controls, but their potential to remodel AT during weight loss is impaired accompanied by decreased glucose tolerance, indicating metabolic inflexibility [29]. Although no obvious morphological changes could be observed in adipose tissue of *Ndst1* AKO mice after a weight loss intervention, their glucose intolerance remained unchanged, indicating that they also suffer from some form of metabolic inflexibility. The latter might also explain why the rFGF1-induced weight loss did not improve glucose homeostasis in *Ext1* AKO and *Ndst1* AKO mice compared to WT controls. This agrees with previous observations exposing that intracerebroventricular FGF1 administration does not improve insulin sensitivity or lower plasma insulin levels [40,53,55]. Intact insulin signaling is required for diabetes remission induced by the central action of FGF1 [40,55].

Although it has been long known that HS, FGF1 and FGFRs interact, new studies emerge that provide more details, showing that the interaction with HS specifically increases thermostability of FGF1 [57]. An interesting aspect is also that loss of mechano-sensor Piezo1, which is important for FGF1 release, leads to similar metabolic phenotypes seen in *Ndst1* AKO mice [33,58]. HSPGs have been shown to be involved in mechano-sensing in endothelial cells [58] and therefore a role for HSPGs in mechano-sensing is also plausible in AT. Therefore, the understanding of the impact of HS *in vivo* is crucial for future drug development. Our study emphasizes that not only the presence, but also the sulfation status of HS is important for the metabolic actions of FGF1. Diabetes has been associated with loss of HS sulfation and quantity in various cell types such as pancreatic islets [59], glomeruli [60], and endothelial cells [61], and cultured adipocytes [62]. We have previously shown that decreased sulfation of macrophage HS leads to increased atherosclerosis, elevated weight gain and insulin resistance upon HFD [43], supporting a concept that natural variations in HS sulfation in certain tissues can account for differences in metabolic dysfunction in obese patients. The 6-*O*-sulfation of HS is particularly important for FGF signaling [63]. Targeted disruption of *Hs6st2* causes obesity and insulin resistance in aged male mice [64]. Also, liver and AT weights are increased in *Hs6st2* full body knock-out mice which is in line with our observations.

Based on our data, correctly sulfated HS evokes protective functions in mature adipocytes under excessive nutrient pressure. HSPGs appear part of a complex regulatory system that maintains adipocyte homeostasis during obesity. Our observations support that FGF1 signaling is disturbed in *Ext1* AKO and *Ndst1* AKO mice, however a limitation of our study is that we cannot rule out that other HS binding factors are contributing to the phenotype. It will be interesting to determine pathways other than the FGF signaling pathway that are affected by the disturbed sulfation pattern and which specific HSPG is primarily responsible for mediating the metabolic benefit in adipocytes. We do not expect that co-administration of recombinant FGF1 with heparin can restore FGF1 signaling in our genetic mouse models. Due to the high affinity of FGF1 for heparin, heparin is likely to act as a competitive inhibitor, sequestering FGF1 and thereby reducing its therapeutic efficacy. Hence, it will be interesting to explore strategies that restore FGF1 signaling in adipose tissue by selectively targeting heparan sulfate-modifying enzymes. Moreover, it will be worthwhile to evaluate if natural variations in adipocyte HS composition and the HSPG repertoire are functionally associated with disease risks, especially in the context of lean, metabolic unhealthy obese, and metabolic healthy obese populations [9].

## CRediT authorship contribution statement

**Chung-Jui Yu:** Conceptualization, Data curation, Funding acquisition, Investigation, Methodology, Software, Writing – review & editing. **Ariane R. Pessentheiner:** Conceptualization, Data curation, Formal analysis, Funding acquisition, Investigation, Methodology, Project administration, Resources, Software, Visualization, Writing – original draft, Writing – review & editing. **Sihao Liu:** Conceptualization, Investigation, Methodology, Writing – review & editing. **Sarah Wax:** Data curation, Investigation, Writing – review & editing. **Marissa L. Maciej-Hulme:** Investigation, Methodology. **Chelsea D. Painter:** Investigation, Methodology. **Bastian Ramms:** Investigation, Methodology. **Daniel R. Sandoval:** Investigation, Methodology. **Anthony Quach:** Investigation, Methodology. **Natalie DeForest:** Investigation, Software, Validation. **G. Michelle Ducasa:** Investigation, Methodology. **Chiara Tognaccini:** Investigation, Methodology. **Caroline Labib:** Investigation, Methodology. **Norah Al-Azzam:** Investigation, Methodology. **Friederike Haumann:** Investigation, Methodology. **Greg Trieger:** Investigation, Methodology. **Patrick Secrest:** Investigation, Methodology. **Amit Majithia:** Conceptualization, Methodology, Supervision. **Aaron C. Petrey:** Conceptualization, Supervision. **Kamil Godula:** Conceptualization, Funding acquisition, Supervision, Writing – review & editing. **Annette R. Atkins:** Conceptualization, Supervision, Writing – review & editing. **Michael Downes:** Conceptualization, Supervision, Writing – review & editing. **Ronald M. Evans:** Conceptualization, Funding acquisition, Supervision, Writing – review & editing. **Philip L.S.M. Gordts:** Data curation, Formal analysis, Funding acquisition, Investigation, Methodology, Project administration, Supervision, Writing – original draft, Writing – review & editing.

## Declaration of competing interest

A.R.A., M.D., and R.M.E. are co-inventors of mutated FGF1 proteins and methods of use and may be entitled to royalties. All other authors have nothing to declare.

## Data Availability

Data will be made available on request.
