# Supplementary material for: Adipocyte heparan sulfate determines type 2 diabetes susceptibility in mice via FGF1-Mediated glucose regulation
Source: Mol Metab. 2025 Oct 8;102:102267. doi: 10.1016/j.molmet.2025.102267 (PMC12555841; doi:10.1016/j.molmet.2025.102267)
Supplement: Multimedia component 1 [file mmc1.pdf]

Adipocyte Heparan Sulfate Determines Type 2 Diabetes Susceptibility in Mice via FGF1-Mediated Glucose Regulation.

Chung-Jui Yu[1,2,3*], Ariane R. Pessentheiner[1,4,*], Sihao Liu[5], Sarah Wax,[6] Marissa L. Maciej-Hulme[1], Chelsea D. Painter[1], Bastian Ramms[1], Daniel R. Sandoval[1], Anthony Quach[1], Natalie DeForest[1], G. Michelle Ducasa[1], Chiara Tognaccini[1], Caroline Labib[1], Norah Al-Azzam[1], Friederike Haumann[1,7], Greg Trieger[6], Patrick Secrest[1], Amit Majithia[1], Aaron C. Petrey[2,3], Kamil Godula[6,8], Annette R Atkins[5], Michael Downes[5], Ronald M. Evans[5], Philip L.S.M. Gordts[1,2,3,8#]

[1]Department of Medicine, Division of Endocrinology and Metabolism, University of California, San Diego, CA, US

[2]Department of Pathology, Division of Microbiology & Immunology, University of Utah, School of Medicine, Salt Lake City, UT, USA

[3]University of Utah Molecular Medicine Program, University of Utah, School of Medicine, Salt Lake City, UT, USA,

[4]Institute of Molecular Biosciences, Division of Biophysics, University of Graz, Austria

[5]Gene Expression Laboratory, Salk Institute for Biological Studies, La Jolla, CA, US

[6]Department of Chemistry and Biochemistry, University of California, San Diego, CA, US

[7]Department of Biochemistry, University Medical Center Hamburg-Eppendorf, Hamburg, Germany

[8]Glycobiology Research and Training Center, University of California, San Diego, La Jolla, CA 92093, USA

***The authors contributed equally.**

#To whom correspondence should be addressed:

Philip L.S.M. Gordts, Department of Pathology, Division of Microbiology & Immunology, University of Utah School of Medicine, Salt Lake City, UT, 84112, Ph: 801/5850704, philip.gordts@path.utah.edu

Extended Data Table S 1and Extended Figures 1-8

EXTENDED DATA TABLE

Extended Data Table 1: Primers used for qPCR analysis.

Gene	Forward primer (5'-3')	Reverse primer (5'-3')
Acc1	TGGTGCAGAGGTACCGAAGTG	CGTAGTGGCCGTTCTGAAACT
Cd36	GAATTAGAACCGGGCCACGTA	CAGCCAGGACTGCACCAATA
F4/80	CTTTGGCTATGGGCTTCCAGTC	GCAAGGAGGACAGAGTTTATCGTG
Fasn	GGAGGTGGTGATAGCCGGTAT	TGGGTAATCCATAGAGCCCAG
Fgf1a	GAAGCCTCCCAGAGCAGACA	GAAGGTTGTGATCTCCCCTTCA
Fgf1b	CCAGCCTGCCAGTTCTTCAG	AAGGTTGTGATCTCCCCTTCAG
Fgfr1c	GCCAGACAACTTGCCGTATG	CTCAAAGGAGACATTCCG
Glut1	GCCCCCAGAAGGTTATTGA	CGTGGTGAGTGTGGTGGATG
Ppara	CCTGAACATCGAGTGTCGAATATG	GCGAATTGCATTGTGTGACATC
Pparg	GCATGGTGCCTTCGCTGA	TGGCATCTCTGTGTCAACCATG
Scd1	TTCTTGCGATACACTCTGGTGC	CGGGATTGAATGTTGTTGTCGT
Srebp1c	GAAACGTGTCAAGAAGTGCAGG	GGAGATGCTATCTCCATGGCA
Tbp, murine	GAAGCTGCGGTACAATTCCAG	CCCCTTGTACCCTTCACCAAT
Tnf	ATTCGAGTGACAAGCCTGTAGC	GGTTGTCTTTGAGATCCATGCC

EXTENDED FIGURES AND LEGENDS

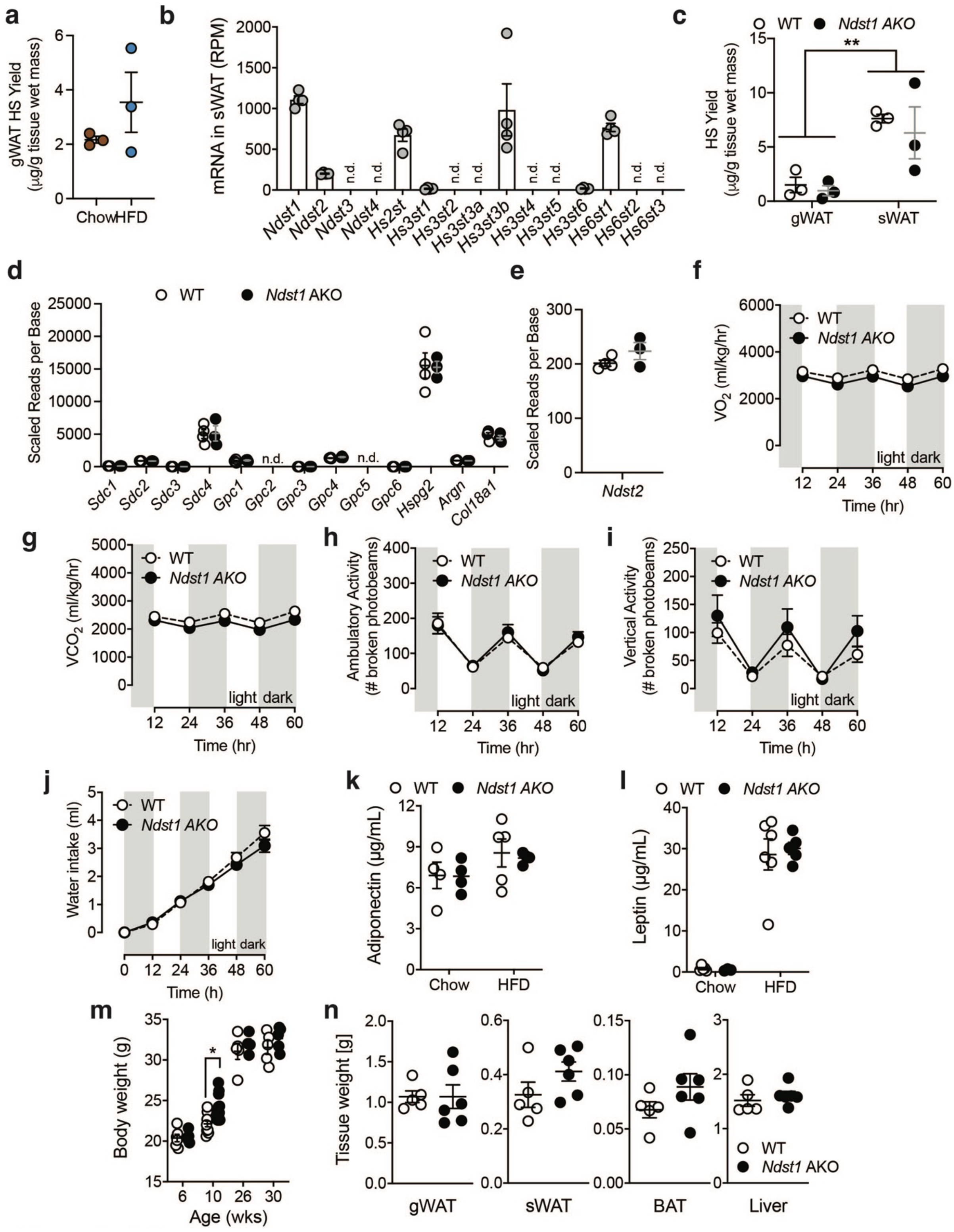

Extended Figure 1: a, Total HS levels in gWAT from mice fed a chow or 60 % HFD for 20 weeks. **b,** mRNA levels of heparan sulfate biosynthetic genes in the AF from sWAT of WT mice. **c,** Total HS levels in gWAT and sWAT from WT and Ndst1 AKO mice fed a 60 % HFD for 18 weeks. **d-e**, mRNA levels of HSPG core proteins (**d**) and Ndst2 (**e**) in adipocytes isolated from sWAT from WT and Ndst1 AKO mice fed a 60 % HFD for 18 weeks. **f-j**, Oxygen consumption, CO2 consumption, ambulatory activity, vertical activity, and water intake levels of *Ndst1* AKO (n = 5) and WT (n = 6) mice fed 60 % HFD for 12 weeks. **k**, Fasting plasma adiponectin levels of *Ndst1* AKO and WT mice fed with CD or 60 % HFD for 12 weeks. **l**, Fasting plasma leptin levels of *Ndst1* AKO and WT mice fed with CD or 60 % HFD for 12 weeks. **m**, Body weight of *Ndst1* AKO and WT mice fed CD. **n**, Tissue weight of 30-week-old *Ndst1* AKO and WT mice fed CD. Data show mean ± s.em., *p < 0.05, vs. WT.

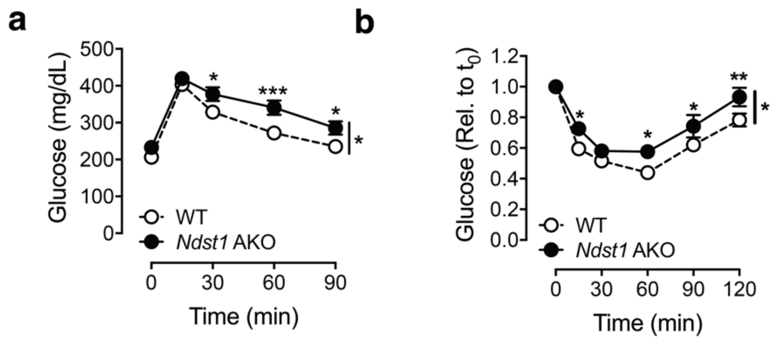

Extended Figure 2: a, GTT on 6-week CD or HFD fed *Ndst1* AKO (n = 23) and WT (n = 18) mice. **b**, ITT on 8-week CD or HFD fed *Ndst1* AKO (n = 15) and WT (n = 14) mice. Data show mean ± s.em., *p < 0.05, **p < 0.01, ***p < 0.001 vs. WT.

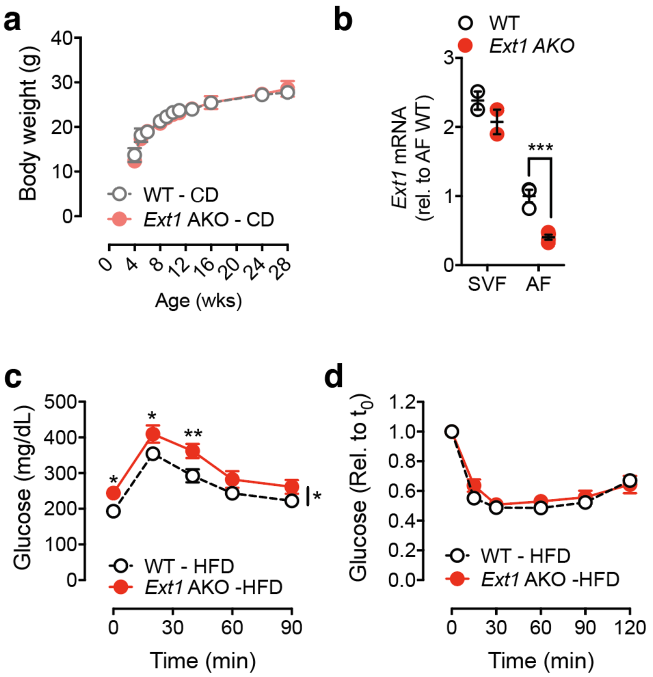

Extended Figure 3: a, Body weight of *Ext1* AKO (n = 6) and WT (n = 6) mice fed with CD. **b**, *Ext1* mRNA levels in SVF and AF from gWAT. **c**, GTT on 8-week CD or HFD fed *Ext1* AKO (n = 6) and WT (n = 8) mice. **d**, ITT on 9-week CD or HFD fed *Ext1* AKO (n = 10) and WT (n = 13) mice. Data show mean ± s.em., $^{*}p < 0.05$, $^{**}p < 0.01$ vs. WT.

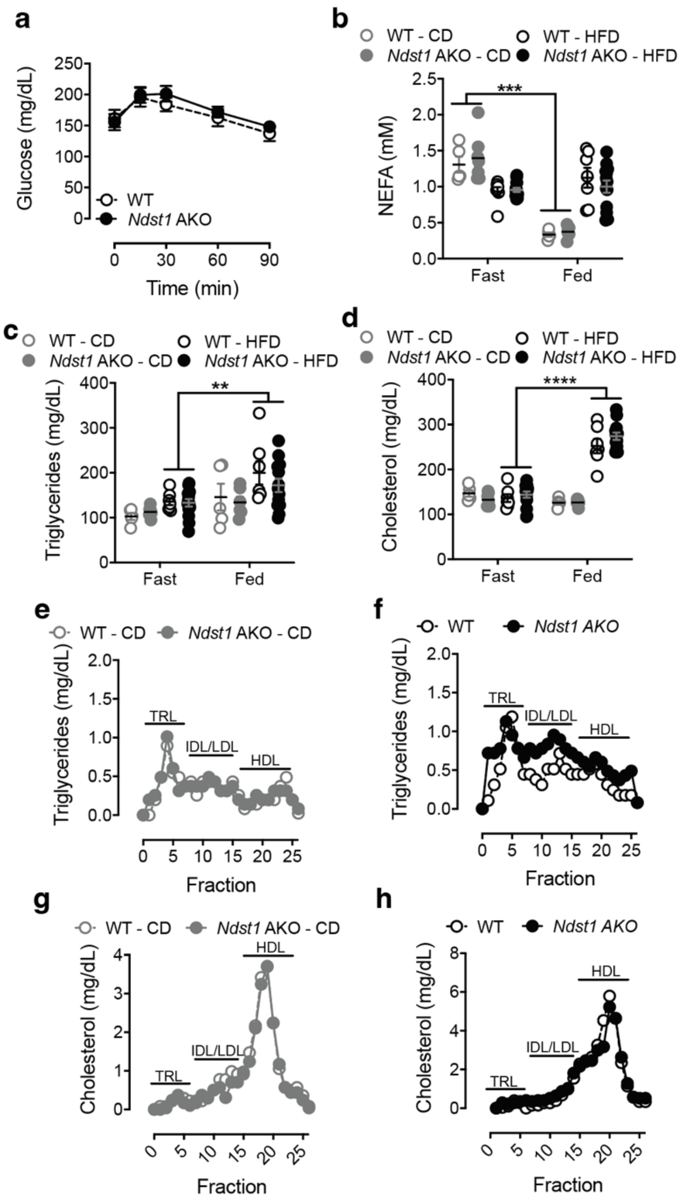

Extended Figure 4: a, PTT on 30-week CD fed *Ndst1* AKO (n = 7) and WT (n = 7) mice. **b-d**, Plasma lipid parameters in fed ad libitum and overnight fasted state in 20-week-old CD or 12-week HFD-fed fed WT and *Ndst1* AKO mice; **b**, NEFA **c**, triglyceride levels, and **d**, cholesterol. **e-h**, Plasma Lipoprotein profiles parameters in 6 hour fasted 20-week-old (**e** and **g**) CD fed or (**f** and **h**) 16-week HFD fed WT and *Ndst1* AKO mice. Data show mean ± s.em., $^{**}p < 0.01$, $^{***}p < 0.001$, $^{****}p < 0.0001$.

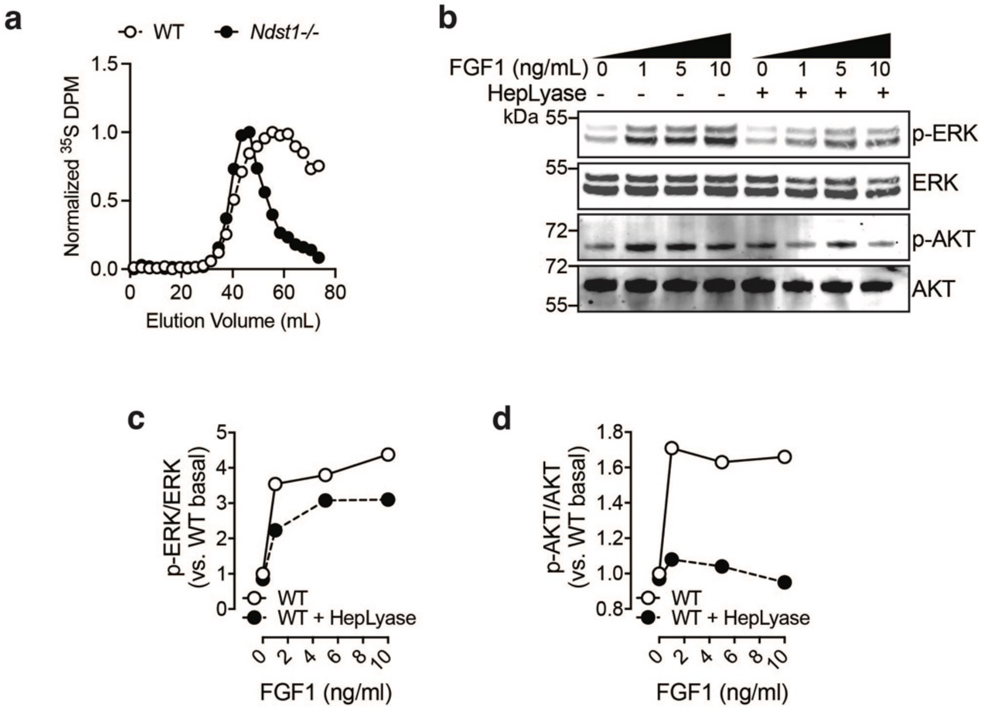

Extended Figure 5: a, HS chain size as determined by size exclusion from WT and Ndst1-/- MEF-derived adipocytes using ^{35}S labeling. **b**, Western blot analysis and quantification of ERK, AKT and p-ERK and p-AKT (p-AKT) after 15 minutes of rFGF1 administration in MEF derived adipocytes without and with Heparin lyase I,II,III (HepLyase) pre-treatment (0.5 U/mL for 30 min; n = 3). **c-d**, Densitometric analysis of Western blot bands for (**b**) p-ERK and (**c**) p-AKT.

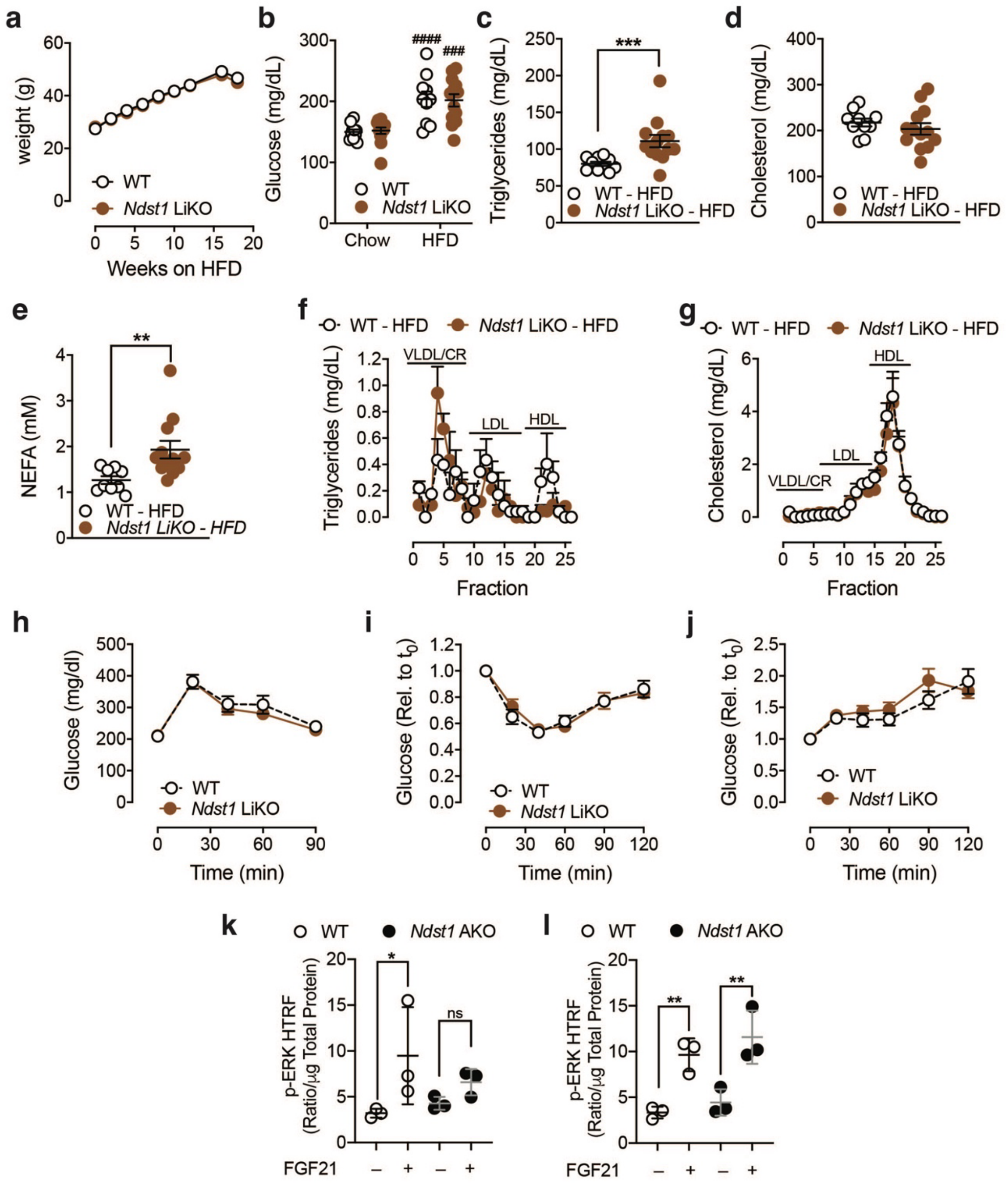

Extended Figure 6: a, Progressive body weights of *Ndst1* LiKO (n = 6) and WT (n = 6) mice maintained on a HFD (60% fat). **b**, Fasting plasma glucose levels of *Ndst1* LiKO and WT mice fed with a HFD for 8 weeks. **c-e**, Plasma lipid parameters in fed ad libitum and overnight fasted state in 12-week HFD-fed WT and *Ndst1* LiKO mice; **c**, triglyceride levels, **d**, cholesterol, and **e**,

NEFA. **f-g**, Plasma Lipoprotein profiles parameters in 6-hour fasted 16-week HFD fed WT and *Ndst1* LiKO mice. **h**, GTT on 16-week CD fed *Ndst*1 AKO and WT mice (n = 14-15 per group). **i**, ITT on 17-week CD fed *Ndst1* AKO and WT mice (n = 5-6 per group). PTT after 16 weeks of HFD in *Ndst1* LiKO (n = 7) and WT (n = 6). **k**, HTRF analysis of ERK phosphorylation in gWAT from FGF21 injected WT and Ndst1 AKO mice fed with a HFD. **l**, HTRF analysis of ERK phosphorylation in sWAT from FGF21 injected WT and Ndst1 AKO mice fed with a HFD. Data show mean ± s.em., $^{**}p < 0.01$, $^{***}p < 0.001$ vs. WT HFD; ###p < 0.001, ####p < 0.0001 vs. CD of same genotype.

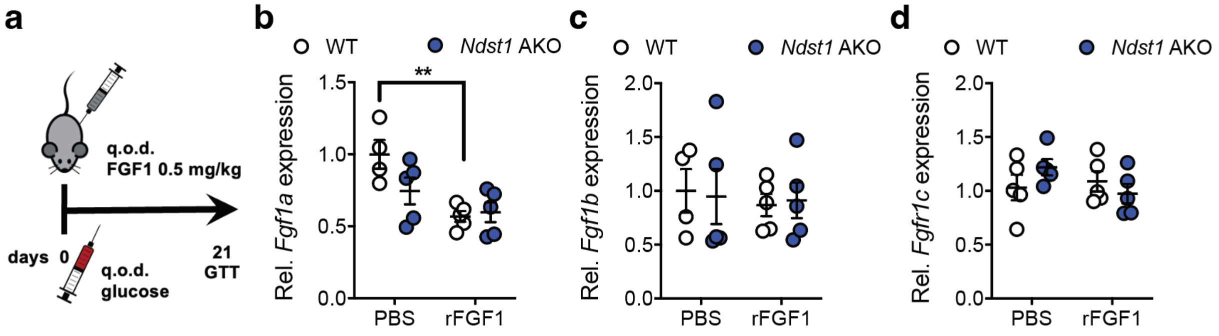

Extended Figure 7: a, Treatment scheme for 3-week every other day i.p injection of rFGF1 (0.5 mg/kg) in WT, Ndst1 AKO and *Ext1* AKO mice after 16 weeks of HFD. **b-d**, mRNA of *Fgf1a*, *Fgf1b* and *Fgfr1c* analyses of eWAT after 21 days of rFGF1 treatment. Data show mean ± s.em., $^{**}p < 0.01$.

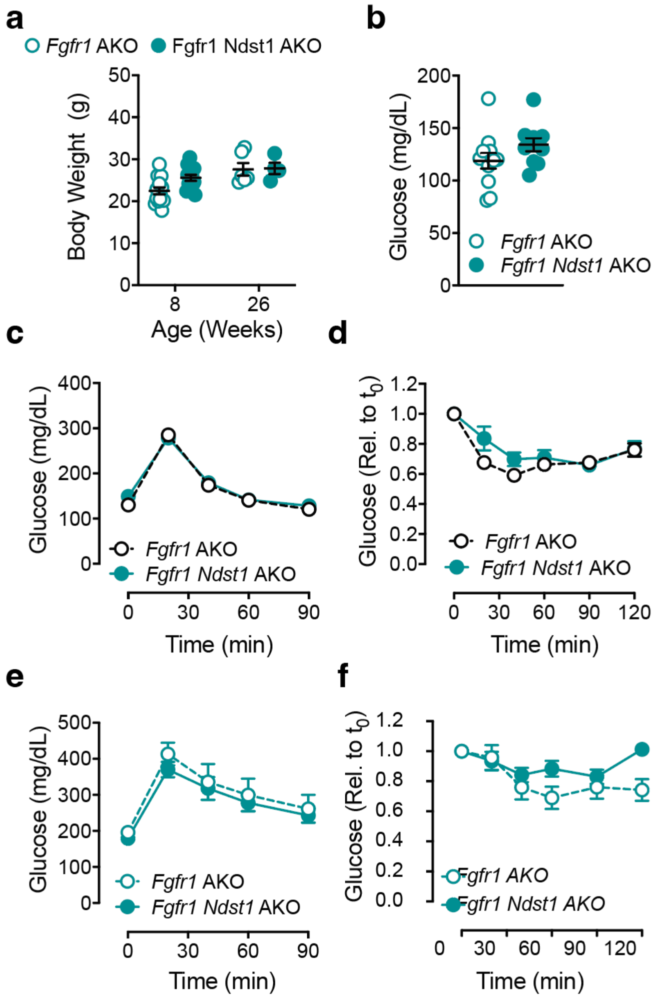

Extended Figure 8: a, Body weight of *Fgfr1 Ndst1* AKO and *Fgfr1* AKO mice fed CD. **b**, Fasting glucose levels of 20-week-old *Fgfr1 Ndst1* AKO and *Fgfr1* AKO mice fed CD. **c**, GTT of CD fed *Fgfr1* AKO (n = 13) and *Fgfr1 Ndst1* AKO (n = 14) mice. **d**, ITT of 9-weeks CD fed *Fgfr1* AKO (n = 10) and *Fgfr1 Ndst1* AKO (n = 9) mice. **e**, GTT of 16-week HFD fed *Fgfr1* AKO (n = 11) and *Fgfr1 Ndst1* AKO (n = 16) mice. **f**, ITT of 17-weeks HFD fed *Fgfr1* AKO (n = 9) and *Fgfr1 Ndst1* AKO (n = 13) mice. Data show mean ± s.em.
